# Isolation, N-glycosylations and Function of a Hyaluronidase-Like Enzyme from the Venom of the Spider *Cupiennius salei*

**DOI:** 10.1371/journal.pone.0143963

**Published:** 2015-12-02

**Authors:** Olivier Biner, Christian Trachsel, Aline Moser, Lukas Kopp, Nicolas Langenegger, Urs Kämpfer, Christoph von Ballmoos, Wolfgang Nentwig, Stefan Schürch, Johann Schaller, Lucia Kuhn-Nentwig

**Affiliations:** 1 Department of Chemistry and Biochemistry, University of Bern, Bern, Switzerland; 2 Functional Genomics Center Zürich, University of Zürich/ETH Zürich, Zürich, Switzerland; 3 Institute of Ecology and Evolution, University of Bern, Bern, Switzerland; University of Roskilde, DENMARK

## Abstract

**Structure of Cupiennius salei venom hyaluronidase:**

Hyaluronidases are important venom components acting as spreading factor of toxic compounds. In several studies this spreading effect was tested on vertebrate tissue. However, data about the spreading activity on invertebrates, the main prey organisms of spiders, are lacking. Here, a hyaluronidase-like enzyme was isolated from the venom of the spider *Cupiennius salei*. The amino acid sequence of the enzyme was determined by cDNA analysis of the venom gland transcriptome and confirmed by protein analysis. Two complex N-linked glycans akin to honey bee hyaluronidase glycosylations, were identified by tandem mass spectrometry. A C-terminal EGF-like domain was identified in spider hyaluronidase using InterPro. The spider hyaluronidase-like enzyme showed maximal activity at acidic pH, between 40–60°C, and 0.2 M KCl. Divalent ions did not enhance HA degradation activity, indicating that they are not recruited for catalysis.

**Function of venom hyaluronidases:**

Besides hyaluronan, the enzyme degrades chondroitin sulfate A, whereas heparan sulfate and dermatan sulfate are not affected. The end products of hyaluronan degradation are tetramers, whereas chondroitin sulfate A is mainly degraded to hexamers. Identification of terminal N-acetylglucosamine or N-acetylgalactosamine at the reducing end of the oligomers identified the enzyme as an endo-β-N-acetyl-D-hexosaminidase hydrolase. The spreading effect of the hyaluronidase-like enzyme on invertebrate tissue was studied by coinjection of the enzyme with the *Cupiennius salei* main neurotoxin CsTx-1 into *Drosophila* flies. The enzyme significantly enhances the neurotoxic activity of CsTx-1. Comparative substrate degradation tests with hyaluronan, chondroitin sulfate A, dermatan sulfate, and heparan sulfate with venoms from 39 spider species from 21 families identified some spider families (Atypidae, Eresidae, Araneidae and Nephilidae) without activity of hyaluronidase-like enzymes. This is interpreted as a loss of this enzyme and fits quite well the current phylogenetic idea on a more isolated position of these families and can perhaps be explained by specialized prey catching techniques.

## Introduction

Hyaluronidases (Hyals) (**EC 3.2.1.35**) are ubiquitously distributed enzymes found in many organisms ranging from bacteria to humans. Eukaryotic enzymes are hydrolases that hydrolyze cellular and extracellular matrix (ECM) polysaccharides from the glycosaminoglycan (GAG) family such as hyaluronan (HA), non-sulfated chondroitin (Ch), and chondroitin sulfate (CS), heparan sulfate (HS) and dermatan sulfate (DS). Bacterial enzymes are lyases which cleave the β-(1,4) linkage in HA, CS and Ch by β-elimination [1].

HA forms very long polymeric structures, which can reach molecular masses up to 6–8 MDa. Chemically, HA consists of repeating glucuronic acid (GlcA) and N-acetylglucosamine (GlcNAc) units [2], whereas Ch is composed of repeating disaccharides of GlcA and N-acetylgalactosamine (GalNAc). The sulfated version of Ch is called CS and different sulfation patterns exist, for instance CS4 is sulfated at the fourth carbon atom of the GalNAc moiety. DS is composed of repeating iduronic acid (IdoA)-GalNAc units, whereas IdoA is mainly sulfated at carbon atom 2 and GalNAc at carbon atom 4. HS is a complex polysaccharide consisting of repeating disaccharides of GlcA or IdoA with glucosamines, which can be sulfated at various positions.[3].

Many small predators use a hunting strategy that relies on highly potent and fast acting venoms to subdue and paralyze their prey. In venoms ranging from vertebrates such as snakes, stonefish, and lizards to invertebrates such as bees, scorpions, and spiders, different Hyals have been identified [4–10]. In general, the assumed function of venom Hyals is the degradation of ECM polysaccharides in the connective tissue of the attacked animal, eventually leading to loss of structural integrity. Therefore, venom Hyals are often described as spreading factors that facilitate the distribution of other venom components through tissues [11], which is important for fast immobilization of prey but also for defense against other predators. The spreading activity of venom Hyals on the ECM of vertebrates has been investigated in several studies [12–15]. *In vivo* dermonecrosis experiments on rabbit skin using recombinant spider venom Hyal (*Loxosceles intermedia*) in combination with recombinant dermonecrotic toxin LiRecDT1 apparently highlight the function of this enzyme as “spreading factor” [16]. However, besides some small vertebrates, the vast majority of spiders predominantly hunt invertebrates.

The GAG arsenal of invertebrates has been reported to consist mainly of HS and CS, with HS as the most frequently occurring GAG [17–19]. HA is ubiquitous in vertebrates [20] and is assumed to be the main substrate for spider venom Hyals [21]. To date, it is supposed that HA is not present in invertebrates such as insects (*Drosophila melanogaster* and *Anopheles stephensi*) and nematodes (*Caenorhabditis elegans*) and no HA synthase gene candidate was found in the genome of *D*. *melanogaster* and *C*. *elegans* [20, 22–25]. Lack of HA in these invertebrate challenges the role of spider venom Hyals as mainly HA hydrolyzing enzymes.

It is hypothesized [20, 26] that Ch appeared before HA during the evolution of invertebrates, because only Ch and HS were identified in *C*. *elegans* [27], and that Hyals might originate from an ancestral chondroitinase. A Hyal-like sequence was identified in *C*. *elegans* and assays with the recombinant protein exhibited depolymerization of Ch and to a lower extent of HA [28]. Additionally, total protein extracts of wild type *C*. *elegans* exhibited depolymerization of Ch and not of HA at acidic and neutral pH conditions [27]. These findings led us to the hypothesis that spreading properties of spider venom Hyals may not only be caused by HA degradation, and that especially in invertebrate-hunting predators Ch or CS degradation may also be important.

In different spider venoms Hyal activity was reported, suggesting a possible wide distribution of this enzyme [29]. However, its substrate specificity was only investigated in a few studies and no information concerning its glycosylation was reported so far. The venom Hyal of the spider *Hippasa partita* and *Brachypelma vagans* were shown to be highly specific for HA, whereas brown spider *Loxosceles intermedia* venom was able to degrade HA as well as CS [13, 21, 30]. No further publications investigating the spreading effect of spider venom Hyals on insects, which are the main prey group of spiders, are available.

Here, we present the isolation of a Hyal-like enzyme from the venom of the Central American spider *Cupiennius salei* (*C*. *salei*) and its enzymatic and structural characterization. The effects of temperature, pH, and mono- and divalent ions on the enzymatic activity were determined, as well as the *K*_m_ and *V*_max_ value. Measurement of the specific activity of CsHyal for HA and CS4 degradation was performed spectrophotometrically via the release of terminal GlcNAc and GalNAc at the reducing end.

Furthermore, the amino acid sequence was elucidated using a combination of cDNA data, Edman degradation, and mass spectrometry, and N-linked glycans were investigated by tandem mass spectrometry. Using the amino acid sequence and homology modeling, a structural model of the *C*. *salei* Hyal-like enzyme (CsHyal) was established.

The spreading effect of CsHyal on invertebrate tissue was investigated by injecting recombinant CsHyal (rCsHyal) into *D*. *melanogaster* and coinjecting rCsHyal with the main *C*. *salei* venom neurotoxin CsTx-1 (Omega-ctenitoxin-Cs1a [P81694]) and the cytolytic acting, small cationic peptide cupiennin 1a (M-ctenitoxin-Cs1a [83619]).

To evaluate the distribution of venom Hyals within spider phylogeny, a total of 39 spider species from 21 families were screened for venom Hyal activity using agarose gel electrophoresis and sequential staining with toluidine blue and Stains-All. Additionally, the substrate specificity of all identified Hyals was tested using HA, CS4, HS, and DS. The end products of HA and CS4 degradation by CsHyal were determined by thin layer chromatography (TLC).

## Materials and Methods

### Spider maintenance and venom collection

Spider breeding and venom collection were conducted as previously described [31]. Venom of *Alopecosa fabrilis*, *Ancylometes rufus*, *C*. *salei*, *Dolomedes okefinokensis*, *Isopeda villosa*, *Linothele megatheloides*, *Phoneutria fera*, *Phoneutria reidyi*, *Polybetes pythagoricus*, *Viridasius fasciatus*, and *Zoropsis spinimana* was obtained from laboratory bred spiders. Venom of *Segestria florentina*, *Steatoda paykulliana*, and *Stegodyphus lineatus* was purchased from Latoxan (France). Venom of *Alopecosa marikovskyi* and *Latrodectus tredecimguttatus* was purchased from FaunaLab (Kazakhstan). *Agelena labyrinthica*, *Araneus diadematus*, *Argiope bruennichi*, *Atypus piceus*, *Callobius claustrarius*, *Larinioides sclopetarius*, *Pisaura mirabilis*, *Meta menardi* and *Tegenaria atrica* were collected in Switzerland, *Filistata insidiatrix* in Italy, *Sericopelma rubronitens* in Panama, *Geolycosa vultuosa* in Hungary, *Nephila pilipes*, and *Oxyopes* sp. in Taiwan, *Eusparassus dufouri*, *Hogna radiata*, *Lycosa hispanica* in Spain, *Araneus angulatus*, *Eresus walckenaerius*, *Lycosa praegrandis*, *Uroctea durandi* in Greece, *Drassodes lapidosus* in Austria, and *Macrothele calpeiana* in Portugal.

To perform these collections no specific permissions were required because we collected on private land of the authors and on publicly accessible land without any protection status such as common land. None of the collected spiders belonged to an endangered or protected species.

### cDNA library of the venom gland of *C*. *salei*

The cDNA library of the *C*. *salei* venom gland transcriptome established in 2011 [32] was used for CsHyal sequence determination, with a total of 17 contigs, comprising 937 sequences encoding CsHyal.

### Structure modeling

The structure of CsHyal was modeled by structure homology modeling using the Phyre2 server [33]. The modeled structure was evaluated by analyzing the φ-, ψ- angles using RAMPAGE [34]. Images from the structures were generated using PyMOL Molecular Graphics System, Version 1.7.0.0 (Schrödinger LLC, South San Francisco, CA, USA).

### Hyal assay

Hyal activity was determined by a turbidometric assay according to di Ferrante [35] with slight modifications. The assay mixture contained 50 μg HA (Fluka), 1 μl (0.4–1 U) bovine Hyal (testes, 400–1000 U/mg, Sigma Aldrich) as internal standard, or 1 μl venom (1:200 diluted with buffer), or an aliquot of the chromatographic fractions in a volume of 0.5 ml, 0.2 M NaAc, 0.15 M NaCl, pH 6. The mixture was incubated for 15 min at 37°C and the enzymatic reaction was stopped by addition of 1 ml 2.5% (w/v) cetyltrimethylammonium bromide in 2% (w/v) NaOH. The absorbance was measured at 400 nm for 10 min against a blank of 0.5 ml assay buffer and 1 ml stop solution. Hyal activity is expressed as turbidity reducing unit (TRU). One TRU is defined as the amount of enzyme needed to hydrolyze 50% HA, taking the absorbance of a tube as 100% to which no Hyal was added.

### Purification of native CsHyal

Purification of native CsHyal was carried out by applying three consecutive steps with a total of 4.6 ml (containing 150–260 mg protein/ml crude venom) *C*. *salei* venom. Firstly, size exclusion chromatography of 300–400 μl crude venom per separation was dissolved in 1.6 ml 0.2 M NH_4_Ac buffer, pH 5.5 and, after centrifugation (20,800 *g*, 10 min, 4°C) the supernatant was separated on a Bio-Gel P-60 column (2 x 100 cm, Bio-Rad) at a flow rate of 0.7 ml/min at room temperature in dissolving buffer. The eluted fractions were monitored for Hyal activity. Secondly, fractions exhibiting Hyal activity were separated using cation exchange chromatography on a Mono S column (HR10/10, GE Healthcare, United Kingdom) equilibrated with 50 mM NH_4_Ac buffer, pH 5. Fractions were eluted within 60 min at a flow rate of 2 ml/min with the same buffer containing 2 M NaCl and monitored for Hyal activity. Thirdly, fractions showing Hyal activity were further separated by RP-HPLC on a Nucleosil 300 butyl column (4.6 x 250 mm, 5 μm, Macherey-Nagel GmbH & Co. KG) equilibrated with 0.1% (v/v) TFA in water. Proteins were eluted with 0.1% (v/v) TFA in acetonitrile (ACN) applying a gradient of 20–100% ACN in 80 min at a flow-rate of 1 ml/min. Fractions with Hyal activity were stored at -20°C until further use.

### SDS-PAGE

SDS-PAGE and silver stain of reduced (2-β-mercaptoethanol) venom and fractions of the purification steps were performed with the Phast-System using homogeneous 12.5% PhastGel (GE Healthcare, United Kingdom). Coomassie blue staining was performed with the eStain 2.0 Protein Staining system (GenScript, USA). Zymograms were performed as described [36] with some modifications using the Phast-System. 10% acrylamide gels containing 8 M urea and 0.01 mg/ml HA were polymerized on the hydrophobic surface of the GelBond®PAG film. Venoms were diluted with sample buffer between 1:5 and 1:100. After electrophoretic separation of spider venoms of different dilutions the polyacrylamide gel was solved from the GelBond®PAG film. Gels were first incubated with 50 mM Tris/Cl pH 7.1, 150 mM NaCl and 2.5% Triton-X-100 twice for 30 min and then washed 3 times with the same buffer without Triton-X-100. Dependent on the separated venoms, gels were incubated 14–20 h at 37° C.

After incubation, gels were washed twice with bidistilled water, followed by an incubation in 3% acetic acid for 30 min. Staining was done with alcian blue 8GX (1% in 3% acetic acid) for 2 h and after destaining in 3% acetic acid, counterstaining with PhastGel® Blue R was performed. As molecular mass standard the LMW electrophoresis calibration kit (GE Healthcare, United Kingdom) was used and staining was done with PhastGel® Blue R.

### Recombinant protein expression

The cDNA sequence encoding the mature Hyal-like enzyme (CsHyal) was optimized for *E*. *coli* codon usage, a 5' *Nde*I and a 3' *Xho*I restriction site were added and the gene was synthesized and subcloned into a pET28a (+) vector (all steps done by GenScript, USA). The recombinant enzyme was expressed as a fusion protein with an N-terminal His_6_-Tag. The expression vector was inserted into BL21 (DE3) chemocompetent *E*. *coli*, spread on LB-agar plates containing 30 μg/ml kanamycin and incubated at 37°C overnight. A single colony was used to inoculate 5 ml LB-kanamycin medium and grown overnight at 37°C, 220 rpm. The culture was diluted 1:200 into 1 l LB-kanamycin medium and incubated at 37°C, 180 rpm until OD_600_ reached 0.4–0.5. Expression was induced by addition of 0.1 mM isopropyl-β-D-thiogalactoside and growth was continued for 4 h at 30°C. Cells were harvested by centrifugation at 4,000 g, 20 min, 4°C, washed with PBS, pH 7.5, and pellets were frozen at - 20°C overnight.

### Purification and refolding of recombinant protein

To the cell pellet 100 ml extraction buffer (100 mM Na_2_HPO_4_, pH 8, 6 M guanidium hydrochloride) was added, cells were lysed for 24 h at 4°C, sonicated using a Vibra-cell VCX 130 probe sonicator (Sonics & Materials, Newtown, CT, USA) at a frequency of 20 kHz for 20 min (5 s on, 10 s off) on ice, and centrifuged at 150,000 g, 4°C, 45 min. The supernatant was further purified by Ni-NTA affinity chromatography under denaturing conditions using Ni Sepharose Fast Flow beads (GE Healthcare, United Kingdom). The column was washed with extraction buffer, pH 6.4, and protein was eluted by decreasing the pH to 4.6. To the eluate 100 mM dithiothreitol (DTT) was added and after incubation for 4h the solution was dialyzed against 100 mM Tris, pH 8, 8 M urea for 24 h at 4°C and concentrated using centrifugal filter devices (MWCO 10 kDa, Vivaspin, Germany) to a concentration of 10 mg/ml. The solution was added dropwise (1:10 ratio) to a refolding buffer (100 mM Tris, pH 8.5, 1 M arginine, 3 mM reduced glutathione, and 1 mM oxidized glutathione) by stirring over 12 h at 4°C, dialyzed for 24 h against PBS buffer, pH 7.4, and concentrated again [37]. Activity of rCsHyal was determined by the HA degradation assay. For bioassays the buffer was exchanged against water by a PD10 column.

### Enzymatic characterization

To determine effects of temperature, pH, mono- and divalent cations on CsHyal activity, and to determine *K*_m_ and *V*_max_, crude venom was diluted until 50% HA was degraded in 15 min under standard assay conditions (0.2 M NaAc, 0.15 M NaCl, pH 6, 37°C). The optimum temperature was examined by incubating venom at different temperatures ranging from 10 to 70°C. Thermostability was investigated by measuring the activity of CsHyal at 4, 25, 37, and 50°C after 1, 3, 6, 12, and 24 h of incubation. The pH-dependent activity was analyzed in standard assay buffer between pH 2–9. The effect of monovalent salts was examined in a 0.2 M NH_4_Ac buffer, pH 5.8, containing 0.2 M NaCl or KCl, respectively. The effect of divalent cations was assayed in 0.2 M NH_4_Ac containing 5 mM of either EDTA, nickel(II) sulfate hexahydrate, copper(II) sulfate, calcium chloride dihydrate, magnesium chloride, manganese(II) sulfate monohydrate, iron(II) sulfate heptahydrate, cobalt(II) chloride hexahydrate, zinc(II) chloride, or sodium molybdate(II) dihydrate (all from Sigma). *K*_m_ and *V*_max_ of crude venom were determined under standard assay conditions with 20, 30, 40, and 50 μg HA and the values were calculated using non-linear regression (GraphPad Prism 6). The velocity was expressed as μg HA degraded per min. Each analysis was conducted with two different aliquots in triplicate and standard deviations were calculated using all six measurements.

### Substrate specificity

10 μg of each GAG standard (HA, CS4, HS, DS; all from Sigma) was incubated overnight with 0.1 μl spider venom in a final volume of 40 μl 0.2 M NH_4_ Ac, 0.15 M NaCl buffer, pH 5.8, at 40°C. After incubation, agarose gel electrophoresis was performed according to Volpi [38] with some minor modifications. Briefly, samples were loaded on 1% agarose gels in 0.04 M Ba(Ac)_2_, pH 5.8, and electrophoresis was performed in 0.05 M 1,2-diaminopropane buffer, pH 9, at 50 mA for 3 h. Gels were immersed overnight in a 0.1% (w/v) cetyltrimethylammonium bromide solution, followed by toluidine blue staining to reveal sulfated GAGs. After a further staining step with Stains-All, sulfated GAGs appeared purple, whilst non-sulfated HA appeared in a bright blue color.

### GAG degradation and carbohydrate analysis

*C*. *salei* venom (1:30) or bovHyal (100 μg) were dissolved in buffer (0.2 M NaAc, pH 6, 0.15 M NaCl) and incubated with HA (100 μg) or CS4 (100 μg) in duplicate, in a total volume of 300 μl, at 38°C for 5 min, 30 min, 1.25 h, 3 h, 5 h, 10 h, 24 h, and 48 h. The obtained degradation products of HA or CS4, oligosaccharides with GlcNAc or GalNAc at the reducing end, were determined by a modification of the Morgan-Elson reaction [39]. The incubation was stopped by adding 50 μl of saturated K_2_B_4_O_7_ x 4 H_2_O solution and directly incubated in a boiling water bath for 3.5 min. Subsequently, the samples were transferred into a water bath at 38°C and after 6 min, 1.5 ml of the 4-(dimethylamino)-benzaldehyde (DMAB; Ehrlich’s reagent) solution was added. After 20 min the samples were cooled down on ice and centrifuged at 4°C, for 30 min at 14,000 g. Measurement of the supernatant was done at 585 nm. Calibration curves were recorded in duplicate with a dilution series of GlcNAc and GalNAc in a concentration range from 0.14 μg/ml to 27.78 μg/ml. As negative controls GlcNAc or GalNAc without enzymes; spider venom and bovHyal without GlcNAc or GalNAc were used. Statistic evaluation of the data was performed with GraphPad Prism 6 (linear regression; comparing of slopes with an ANCOVA equivalent).

### Thin-layer chromatography (TLC)

Separation of degradation products of HA or CS4 was achieved by TLC on silica gel 60 (aluminium sheets, 5x10 cm, Merck) [40]. Briefly, *C*. *salei* venom (1:30) or bovHyal (100 μg) were dissolved in buffer (0.2 M NaAc, pH 6, 0.15 M NaCl) and incubated with HA (100 μg) or CS4 (100 μg) in a total volume of 300 μl at 38°C for 5 min,1.25 h, and 48 h. The degradation was stopped by placing the samples for 5 min into a boiling water bath. After cooling down in ice water, the samples were centrifuged at 4°C for 30 min at 14,000 g and 10 μl supernatant was placed onto the silica gel. The solvent system was isopropanol-water (66:34), containing 0.05 M NaCl. Degradation products of HA and CS4 were stained with the orcinol-H_2_SO_4_ method [41]. Identification of degradation products was based on oligo-HA of different sizes. HA-5 (non-reducing-GlcNAc-[GlcA-GlcNAc]-[GlcA-GlcNAc]-reducing end), HA-7 (non-reducing-GlcNAc-[GlcA-GlcNAc]-[GlcA-GlcNAc]-[GlcA-GlcNAc]-reducing end), and HA-9 (non-reducing-GlcNAc-[GlcA-GlcNAc]-[GlcA-GlcNAc]-[GlcA-GlcNAc]-[GlcA-GlcNAc]-reducing end) oligosaccharides were purchased from Hyalose, USA. Negative controls were GlcNAc or GalNAc without enzymes; spider venom and bovHyal without GlcNAc or GalNAc.

### Edman Degradation and Amino Acid Analysis

N-terminal sequence analysis was carried out according to Edman on a Procise 492 cLC protein sequenator (Applied Biosystems). The released phenylthiohydantoin amino acids were separated on-line by RP-HPLC and monitored at 269 nm. Amino acid analysis was performed as reported [42].

### Mass spectrometry

For MALDI-TOF-MS an Autoflex III Smartbeam mass spectrometer (Bruker Daltonics) was used. Acquisition of mass spectra was performed with FlexControl and spectra analysis, annotation, and processing with FlexAnalysis software. Sample preparation was carried out directly on a steel target plate by mixing 1 μl of sinapinic acid matrix solution with 1 μl of acidic sample solution. After air drying, samples were analyzed in the linear positive-ion mode between 10–100 kDa.

For LC-MS/MS analysis three aliquots of 5–10 μg purified native CsHyal were dissolved in 100 mM NH_4_HCO_3_ buffer, pH 8, and reduced with 5 mM DTT for 45 min at 50°C. Reduced Cys was modified with 20 mM iodoacetamide at room temperature for 60 min in the dark. Modification was stopped by adding 10 mM DTT and incubation for 15 min. Enzymatic cleavages were performed with trypsin, trypsin/Lys-C (both Promega), and Glu-C (Roche Diagnostics), at an enzyme to substrate ratio of 1:50. All digests were incubated overnight at 37°C and 700 rpm and stopped by adding TFA to a final concentration of 0.5%. The samples were desalted on ZipTip C18 tips (Merck Millipore) applying the manufacturer’s protocol with slight modifications. Desalted samples were dried completely in a vacuum centrifuge and reconstituted with 20 μl of 3% ACN, 0.1% formic acid. 5–10 μL of each peptide solution was analyzed on a Q-Exactive mass spectrometer (Thermo Scientific) coupled to an EASY-nLC1000 (Thermo Scientific). Instrument parameters are based on the “sensitive” method published by Kelstrup et al. [43] with slight modifications. Full scan MS spectra were acquired in profile mode from 300–1700 m/z with an automatic gain control target of 3e6, an Orbitrap resolution of 70’000 (at 200 m/z), and a maximum injection time of 120 ms. The 12 most intense multiply charged (z = +2 to +8) precursor ions from each full scan were selected for higher-energy collisional dissociation fragmentation with a normalized collision energy of 30 (arbitrary unit). Generated fragment ions were scanned with an Orbitrap resolution of 35’000 (at 200 m/z), an automatic gain control value of 5e4, and a maximum injection time of 120 ms. The isolation window for precursor ions was set to 2.0 m/z and the underfill ratio was at 1% (refereeing to an intensity of 4.2e3). Each fragmented precursor ion was set onto the dynamic exclusion list for 90 s. Peptide separation was achieved by RP-HPLC on an in-house packed C18 column (150 mm x 75 μm, 1.9 μm, C-18 AQ, 120 Ǻ, Dr. Maisch GmbH, Germany). Samples were loaded with maximum speed at a pressure restriction of 400 bar and separated with a linear gradient from 3–25% solvent B (0.1% formic acid in ACN, Biosolve BV, Netherlands) in solvent A (0.1% formic acid in H_2_O, Biosolve BV, Netherlands) at a flow rate of 250 nl/min. The column was washed after the separation by flushing with 95% solvent B for 10 min and automatically equilibrated prior to the next injection.

### MS/MS data analysis

LC-MS/MS data were analyzed with Proteome Discoverer (version: 1.4.0.288; DBVersion: 79) in combination with MASCOT (version: 2.4) against the in house built fgcz_arachnida_20140513.fasta database (containing all Arachnida sequences in UniProtKB as of 13.02.2013 supplemented with the aa sequence of CsHyal and 261 known contaminants). The search parameters were: max. missed cleavage = 3, precursor ion tolerance = 10 ppm, fragment ion tolerance = 50 mmu, fixed modification = carboxyamidomethyl (C), variable modification = oxidation (M)/amidation (protein C-terminus). Searches were performed using the MASCOT decoy option and results were filtered with percolator. Only peptides having a percolator score of q < 0.01 were considered as trustworthy. The mass spectrometry proteomics data were deposited to the ProteomeXchange Consortium [PRIDE] via the PRIDE partner repository with the dataset identifier PXD002489 [44].

Glycopeptide analysis was performed with Thermo Xcalibur Qual Browser (version 3.0.63, Thermo Scientific) by manually screening acquired MS/MS spectra for diagnostic glycopeptide marker ions (138.05 m/z, 204.087 m/z, 366.14 m/z) followed by manual assignment of fragment ion spectra. Annotation of assigned spectra was performed with R using the protViz CRAN package. Semi-quantitative information about the distribution of the different identified glycosylation subform within the corresponding glycosylation site was extracted by MS1 filtering [45] using the Skyline daily software (version.2.5.9.6662, 64 bit).

### Bioassays

Bioassays were performed with 1–10 days old female *D*. *melanogaster* as described by Escoubas et al. [46]. Prior to injection, flies were immobilized on ice. Per fly, a dose of 0.05 μl sample dissolved in 0.1 M NH_4_Ac, pH 6.1 was injected laterally into the mesothorax. The mortality rate (or complete paralysis) was assessed after 1, 5, 16, and 24 h. Per treatment, six groups of ten animals each were injected. As control, flies were injected with buffer only and rCsHyal at a non-toxic concentration of 0.42 TRU/mg fly. To assess synergistic effects between rCsHyal and CsTx-1, rCsHyal was coinjected at non-toxic concentrations, 0.42 TRU/mg fly, with 0.40 pmol CsTx-1/mg fly, respectively. As control for the toxicity of CsTx-1 alone, 0.40 pmol CsTx-1/mg fly, was injected separately. Additionally, combinatorial injections of rCsHyal at non-toxic concentrations (0.42 TRU/mg fly), with 7.45 pmol Cu 1a/mg fly were performed. As control for the toxicity of Cu 1a alone, 7.45 pmol Cu 1a/mg fly, was injected separately. CsTx-1 and Cu 1a were purified from the venom of *C*. *salei* as described elsewhere [31, 47]. Results from the combinatorial injection experiments were analyzed using two-way ANOVA and Bonferroni post-tests to compare replicate means by row (GraphPad Prism 6).

## Results

### Purification of native CsHyal

**Fig 1:**
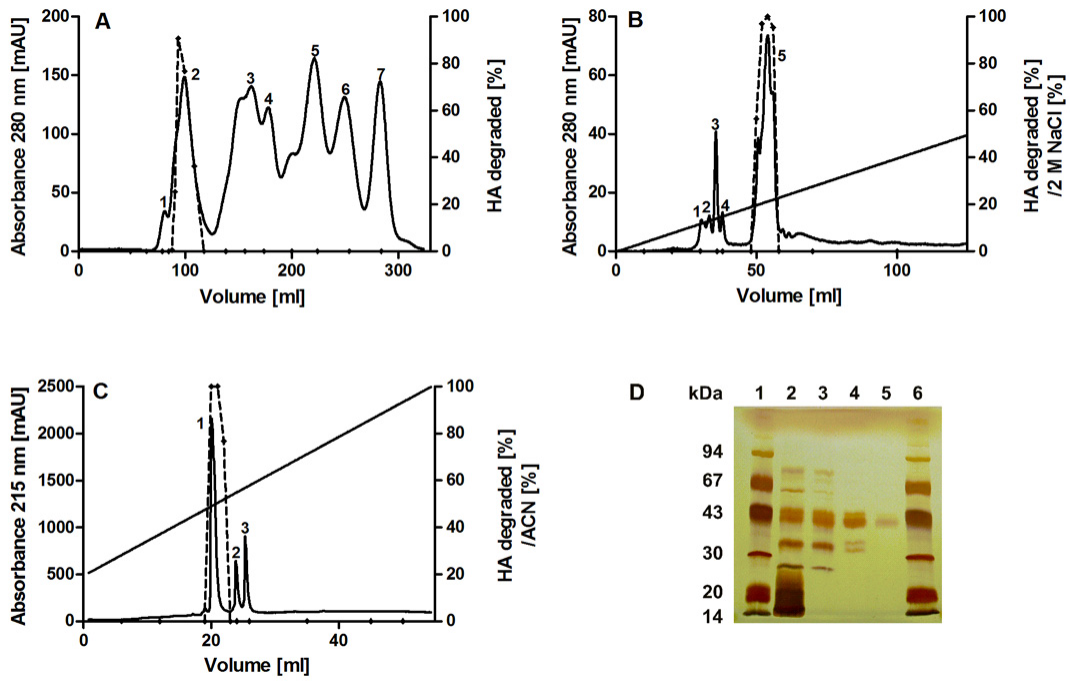
Purification of native CsHyal. (A) Size exclusion chromatography of 400 μl crude venom from *C*. *salei* on a Bio-Gel P-60 column (2 x 100 cm, flow-rate: 0.7 ml/min) in 0.2 M NH_4_Ac buffer, pH 5.5. (B) Cation exchange chromatography of Hyal-active fractions on a MonoS column (HR 10/10, flow-rate: 2 ml/min) with 50 mM NH_4_Ac buffer, pH 5. Fractions were eluted in 60 min by applying a gradient of 0–50% of the same buffer containing 2 M NaCl. (C) RP-HPLC of Hyal-active fractions on a Nucleosil 300 butyl column (4.6 x 250 mm, 5 μm, flow-rate: 1 ml/min) equilibrated with 0.1% TFA (v/v) in water. Fractions were eluted in 80 min by applying a gradient of 20–100% solvent B 0.1% TFA (v/v) in ACN. (D) Purity of chromatographic fractions after the different separation steps was controlled by SDS PAGE (12.5%). Lanes: 1 and 6, low molecular mass markers (14.4–94 kDa, GE Healthcare); 2, crude venom; 3, gel filtration fraction 2; 4, MonoS fraction 5; 5, RP-HPLC fraction 1.

A three step protocol was established to isolate native CsHyal from the crude venom of *C*. *salei*. Firstly, size exclusion chromatography on a Bio-Gel P-60 column separated the crude venom into seven fractions, of which only fraction 2 showed HA degradation activity with a specific activity of 6309 TRU/mg (Fig 1A, Table 1). This equals a 15-fold purification compared with whole venom extract. Secondly, the Hyal-active fraction was further separated by cation exchange chromatography on a MonoS column, and only fraction 5 showed Hyal activity with a specific activity of 10,681 TRU/mg and 26-fold purification over crude venom (Fig 1B, Table 1). Thirdly, rechromatography of fraction 5 by RP-HPLC on a butyl column decreased the specific activity 3.4 times to 3,115 TRU/mg (Table 1), but resulted in a pure Hyal-like enzyme (fraction 1, Fig 1C) corresponding to a mass of 46,288 Da [M+H]^+1^, as measured by MALDI-TOF-MS (S1 Fig). SDS-polyacrylamide gel electrophoresis (Fig 1D), N-terminal sequence analysis, and amino acid analysis confirmed high purity of the isolated enzyme. Overall, different purification cycles of more than 5 ml crude *C*. *salei* venom yielded between 0.06–0.43 μg pure CsHyal/μl venom, equally to a concentration of 1.3–9.3 μM.

**Table 1:** Purification overview of native CsHyal. 320 μl venom of *C*. *salei* were separated by successive chromatography (Fig 1) and the specific activity of chromatographic fractions (Fr.) was determined. All data are given ± SD.

Step	Sample	Total activity [TRU]	Total protein [mg]	Specific activity [TRU/mg]	Fold purification
Venom	Venom	33,945 ± 9	83 ± 4	408 ± 0.5	1
Size-exclusion chromatography (Fig 1A)	Fr. 2	7,232 ± 2	1.15 ± 0.06	6,309 ± 10	15
Cation exchange chromatography (Fig 1B)	Fr. 5	4,959 ± 5	0.46 ± 0.03	10,681 ± 63	26
RP-HPLC (Fig 1C)	Fr. 1	442 ± 2	0.14 ± 0.005	3,115 ± 48	8

### cDNA structure of CsHyal

**Fig 2:**
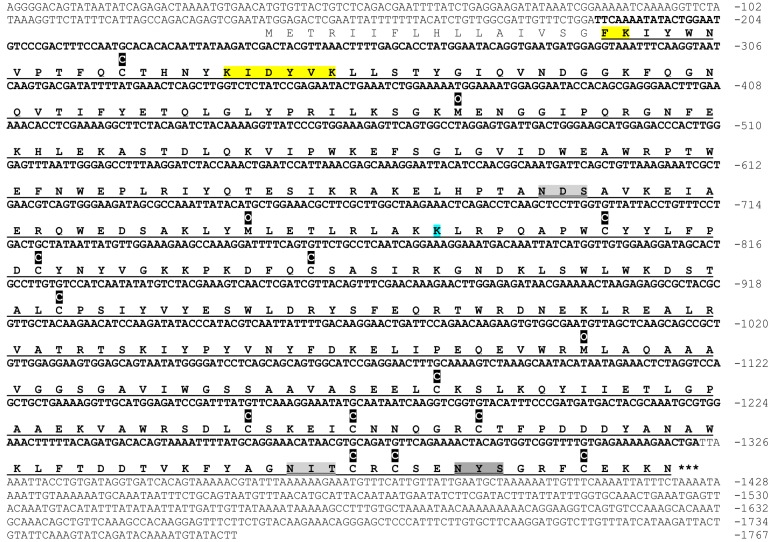
cDNA and amino acid sequence of CsHyal. The deduced amino acid sequence is presented below the nucleotide sequence. The amino acid sequence of the mature protein is *underlined* and the corresponding nucleotide sequence is in *bold*. The *gray doubly underlined* sequences correspond to experimentally determined glycosylation sites and the *dark-gray doubly underlined* sequence to a tentative glycosylation site. The *asterisks* mark the stop codon. The sequence determined by tandem mass spectrometry results in a merged sequence coverage of the trypsin, GluC, and trypsin/LysC cleavage of purified CsHyal. The total sequence coverage including the signal peptide is 94.2%. Amino acids (not colored) have percolator scores with q < 0.01, those in blue are not trustworthy and not identified amino acid residues are colored in yellow. The letter C (black boxed) above the sequence stands for carboxyamidomethylcysteine and O (black boxed) for oxidation of methionine. The analysis of MS data sets was done with Proteome Discoverer Software (version 1.4.0.288, Thermo Fisher Scientific, CA, USA).

Using the *C*. *salei* venom gland cDNA library [32], it was possible to elucidate the cDNA sequence encoding for CsHyal. The cDNA structure starts with a 127 bps long 5’-UTR, followed by an 1182 bps ORF and 444 bps long 3’-UTR. The predicted protein sequence starts with a 16 amino acid long signal peptide followed by the mature protein composed of 378 amino acids (Fig 2). The calculated average mass of the mature protein is 43,939 Da with a theoretical pI of 8.6. Three tentative N-glycosylation sites were identified at positions N134, N360, and N368. The sequence is deposited at the European Nucleotide Archive ENA (Accession#: LN878098).

### Structural characterization of native CsHyal

**Fig 3:**
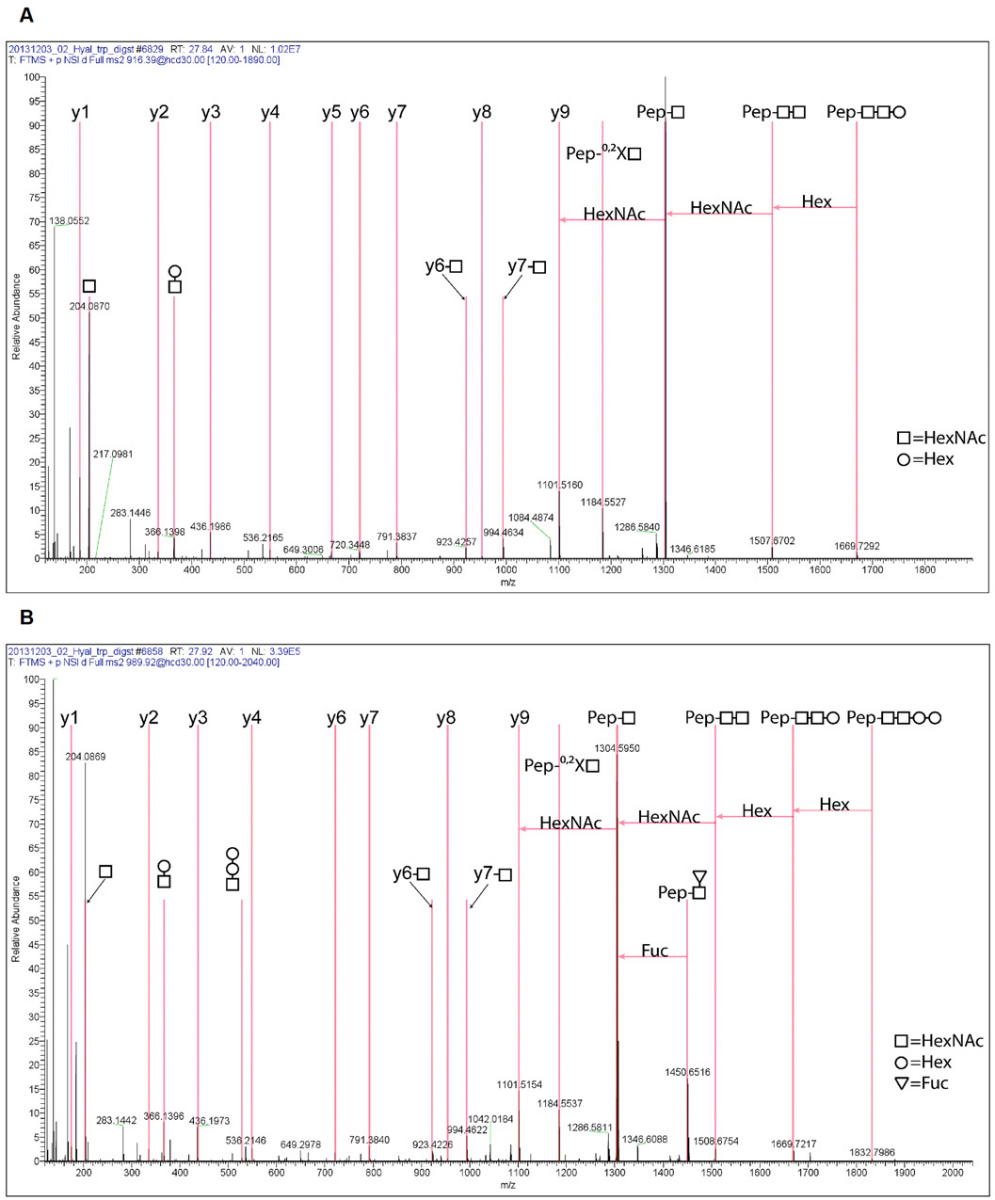
Comparison of the tandem mass spectra of a fucosylated and non-fucosylated glycopeptide originating from the glycosylation site at N360. (A) Positive ESI fragment ion spectrum of the peptide FYAGNITCR modified with the N-glycan of the composition (HexNAc)_2_(Hex)_2_. The [M+2H]^2+^ precursor at 916.394 m/z was fragmented with HCD at a normalized collision energy of 30. The peptide y ion series as well as the relevant fragment ions for the identification of the N-glycan are indicated. (B) Positive ESI fragment ion spectrum of the peptide FYAGNITCR modified with the N-glycan of the composition (HexNAc)_2_(Hex)_2_(Fuc). The [M+2H]^2+^ precursor at 989.423 m/z was fragmented with HCD at a normalized collision energy of 30. The peptide y ion series as well as the relevant fragment ions for the identification of the N-glycan are indicated.

Purified native CsHyal was reduced, alkylated, and cleaved with either trypsin, GluC, or trypsin/LysC. The peptide mixtures were analyzed by tandem mass spectrometry. The resulting MS/MS data were searched against a FASTA database using MASCOT, filtered by percolator, and the results were mapped onto the cDNA sequence of CsHyal. Combination of the results of all three digestions leads to a sequence coverage of 94.2% including the signal sequence (Fig 2). The N-terminal sequence of native CsHyal was determined by 14 cycles of Edman degradation, in agreement with the cDNA sequence data. The MS/MS data sets of the CsHyal proteolytic digests were further used to analyze N-linked glycans. Two glycosylation sites at positions N134 and N360 (Fig 2) were identified revealing a heterogeneous mixture of various glycoforms, whereas no evidence for glycosylation at the third tentative glycosylation site N368 was found. An example of each, a fucosylated and non fucosylated glycopeptide fragment ion spectrum from the glycosylation site at N360, is depicted in Fig 3A and 3B. Additionally, the full set of annotated fragment ion spectra is given in S1 Table and S2 Fig.

**Fig 4:**
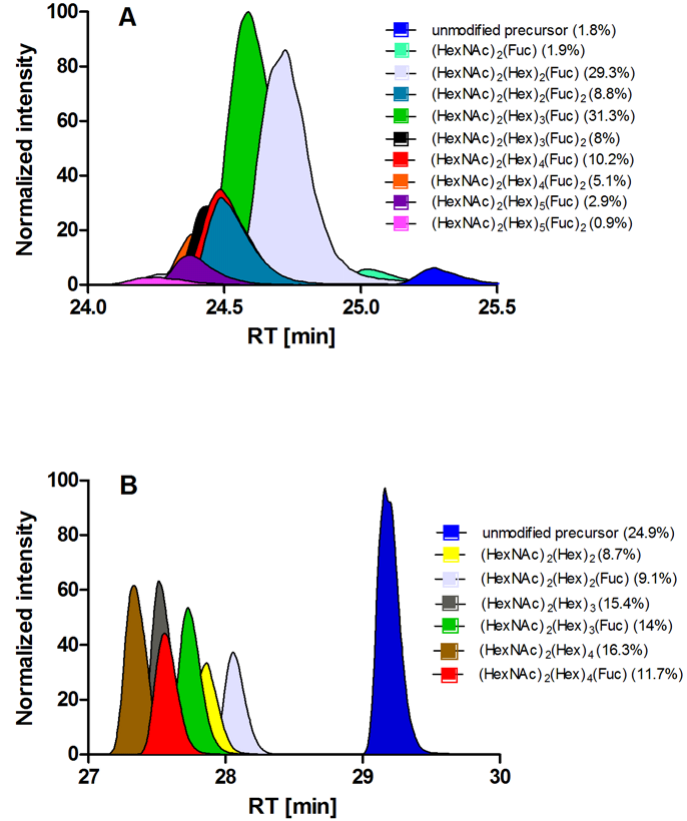
Semi-quantitative extraction of glycopeptide abundances by MS 1 filtering. The precursor ion mass of the identified glycopeptides was used to extract the chromatographic area. The percental distribution of the abundances of the different N-glycans within the two glycosylation sites was calculated. Plot y-axis were normalized to the most abundant value in the graph. N-glycans exhibiting a normalized area of < 2% are not shown in this plot. (A) Area plot of the different glycopeptides on glycosylation site N134. (B) Area plot of the different glycopeptide subforms on glycosylation site N360.

Position N134 is almost fully glycosylated (98.2%) and nine different N-glycan structures with the core structure HexNAc_2_Hex_0-5_Fuc_1-2_ were identified. The three most abundant identified structures were: HexNAc_2_Hex_2_Fuc (29.3%), HexNAc_2_Hex_3_Fuc (31.1%), and HexNAc_2_Hex_4_Fuc (10.2%) (Fig 4A, Table 2). Position N360 is glycosylated to a lower extent (75.1%) and showed six different N-linked glycans with the structure HexNAc_2_Hex_2-4_Fuc_0-1_ (Fig 4B, Table 2). In contrast to position N134, no difucosylated N-linked glycans were identified at position N360.

**Table 2:** Overview of identified glycopeptides. Tabular summary of the manually assigned glycopeptides from the MS/MS data gained by proteolytic digests of native CsHyal and subsequent tandem mass spectrometry. The table gives the deviation in ppm between the theoretically calculated neutral mass and neutral mass based on the experimental data. C* in the peptide sequence corresponds to carboxyamidomethylcysteine. All compositions of the glycan structures were calculated based on the difference between the experimental neutral mass and the peptide neutral mass. Only the glycans with exceed 2% for a given glycosylation site are reported in this Table. The full set of all identified glycan structures is presented in supporting information (S1 Table, S2 Fig).

	exp. neutral mass (Da)	calc. neutral mass (Da)	deviation (ppm)	peptide sequence	peptide neutral mass (Da)	composition of glycan structure	glycosylation neutral mass (Da)
corresponds	2630,289	2630,290	0,27	AKELHPTANDSAVKEIAER	2078,073	(HexNAc)_2_(Fuc)	552,217
to	2954,397	2954,395	0,59	AKELHPTANDSAVKEIAER	2078,073	(HexNAc)_2_(Hex)_2_(Fuc)	876,322
Fig 4A	3100,457	3100,453	1,37	AKELHPTANDSAVKEIAER	2078,073	(HexNAc)_2_(Hex)_2_(Fuc)_2_	1022,380
	3116,452	3116,448	1,23	AKELHPTANDSAVKEIAER	2078,073	(HexNAc)_2_(Hex)_3_(Fuc)	1038,375
	3262,509	3262,506	1,07	AKELHPTANDSAVKEIAER	2078,073	(HexNAc)_2_(Hex)_3_(Fuc)_2_	1184,433
	3278,505	3278,501	1,25	AKELHPTANDSAVKEIAER	2078,073	(HexNAc)_2_(Hex)_4_(Fuc)	1200,428
	3424,562	3424,559	1,08	AKELHPTANDSAVKEIAER	2078,073	(HexNAc)_2_(Hex)_4_(Fuc)_2_	1346,486
	3440,554	3440,554	0,23	AKELHPTANDSAVKEIAER	2078,073	(HexNAc)_2_(Hex)_5_(Fuc)	1362,481
	3586,612	3586,612	0,23	AKELHPTANDSAVKEIAER	2078,073	(HexNAc)_2_(Hex)_5_(Fuc)_2_	1508,539
corresponds	1830,772	1830,771	0,80	FYAGNITC*R	1100,507	(HexNAc)_2_(Hex)_2_	730,264
to	1976,829	1976,829	0,07	FYAGNITC*R	1100,507	(HexNAc)_2_(Hex)_2_(Fuc)	876,322
Fig 4B	1992,823	1992,825	0,98	FYAGNITC*R	1100,507	(HexNAc)_2_(Hex)_3_	892,317
	2138,883	2138,881	1,00	FYAGNITC*R	1100,507	(HexNAc)_2_(Hex)_3_(Fuc)	1038,374
	2154,876	2154,875	0,34	FYAGNITC*R	1100,507	(HexNAc)_2_(Hex)_4_	1054,368
	2300,934	2300,933	0,37	FYAGNITC*R	1100,507	(HexNAc)_2_(Hex)_4_(Fuc)	1200,426

### Structure modeling

The CsHyal structure was modeled on the basis of the crystal structure of human Hyal-1 [48]. The Ramachandran plot of the φ- and ψ- angle of the input and output structure were very similar with 95.7% versus 94.7% of the residues in the favored region, 4.1% versus 4.5% of the residues in the allowed region, and 0.2% versus 0.8% in the outlier region (human versus *C*. *salei*).

**Fig 5:**
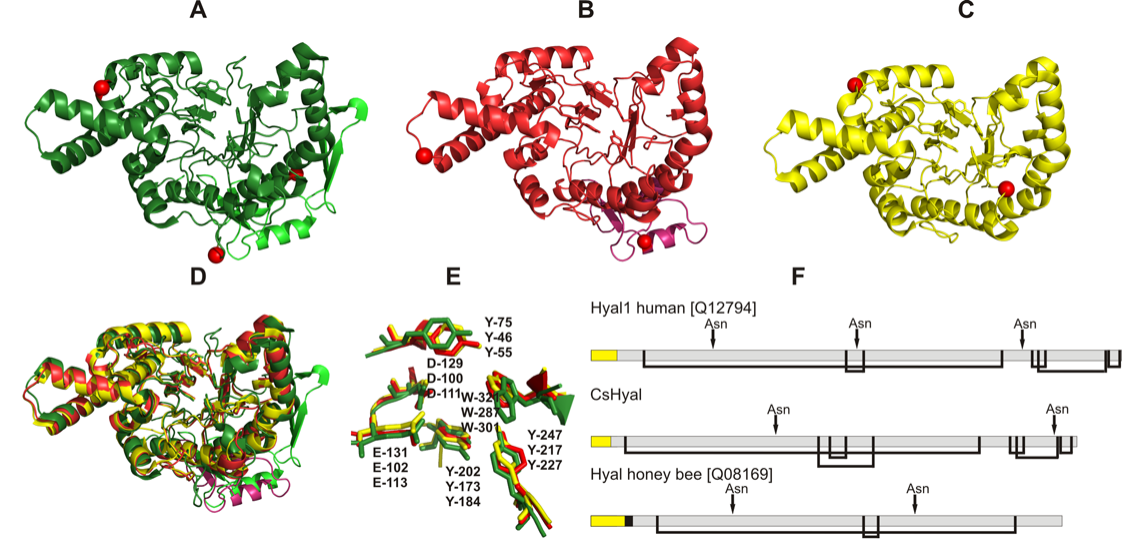
Structural comparison of different Hyals. The residues of the active sites are shown as sticks and experimentally determined N-linked glycosylation sites are indicated by *red* spheres (A-C). The Hyal domain is colored in green and the EGF-like domain in bright green (A, human Hyal-1), the Hyal domain in red and the EGF-like domain in pink (B, *C*. *salei*), and the Hyal domain in yellow (C, bee venom Hyal). All structures were drawn using PyMOL. (A) Crystal structure of human Hyal-1 (PDB:2PE4) determined by Chao et al. [48]. (B) Structure of CsHyal modeled using the human Hyal-1 structure as template. (C) Crystal structure of bee venom Hyal (PDB:1FCQ) determined by Markovic-Housley et al. [49]. (D) Overlay/alignment of the structures of human Hyal-1 (green), *C*. *salei* Hyal (red) and bee venom Hyal (yellow). Active site residues are shown as sticks and EGF-like domains are colored in green and pink, respectively. (E) Zoom of the structures in D shows the active site residues. (F) Disulfide bridge arrangement and glycosylation sites of human, *C*. *salei* and honey bee Hyal. Yellow boxes refer to signal peptides, black box to propeptide and gray boxes to mature proteins.

Sequence alignment (S4 Fig) of CsHyal with human Hyal-1 (identity of 33.9%) and bee venom Hyal (identity of 34.4%) [49] exhibit identical active site residues with Y75, D129, E131, Y202, Y247, and W321 (nomenclature according to human Hyal-1 sequence) as seen in Fig 5E. For CsHyal, an EGF-like domain (S4 Fig) was identified using InterPro (http://www.ebi.ac.uk/Tools/pfa/iprscan5/).

Further, the overall structure of all three Hyals resembles a reduced TIM barrel fold with 7 β-strands [49]. The substrate binding groove is formed by the β-strands of the reduced TIM-barrel. The major difference within the set of the three structures is the missing EGF-like domain in case of honey bee venom Hyal and the different sites and numbers of N-linked glycosylations (Fig 5A–5C). In contrast to human Hyal-1, CsHyal exhibits a further disulfide bridge Cys_171_-Cys_213_ in the neighborhood of Cys_178_-Cys_191_ (corresponding to the disulfide bridge of human Hyal-1, Cys_207_-Cys_221_ [48] (Fig 5F).

### Enzymatic activity characterization of CsHyal

**Fig 6:**
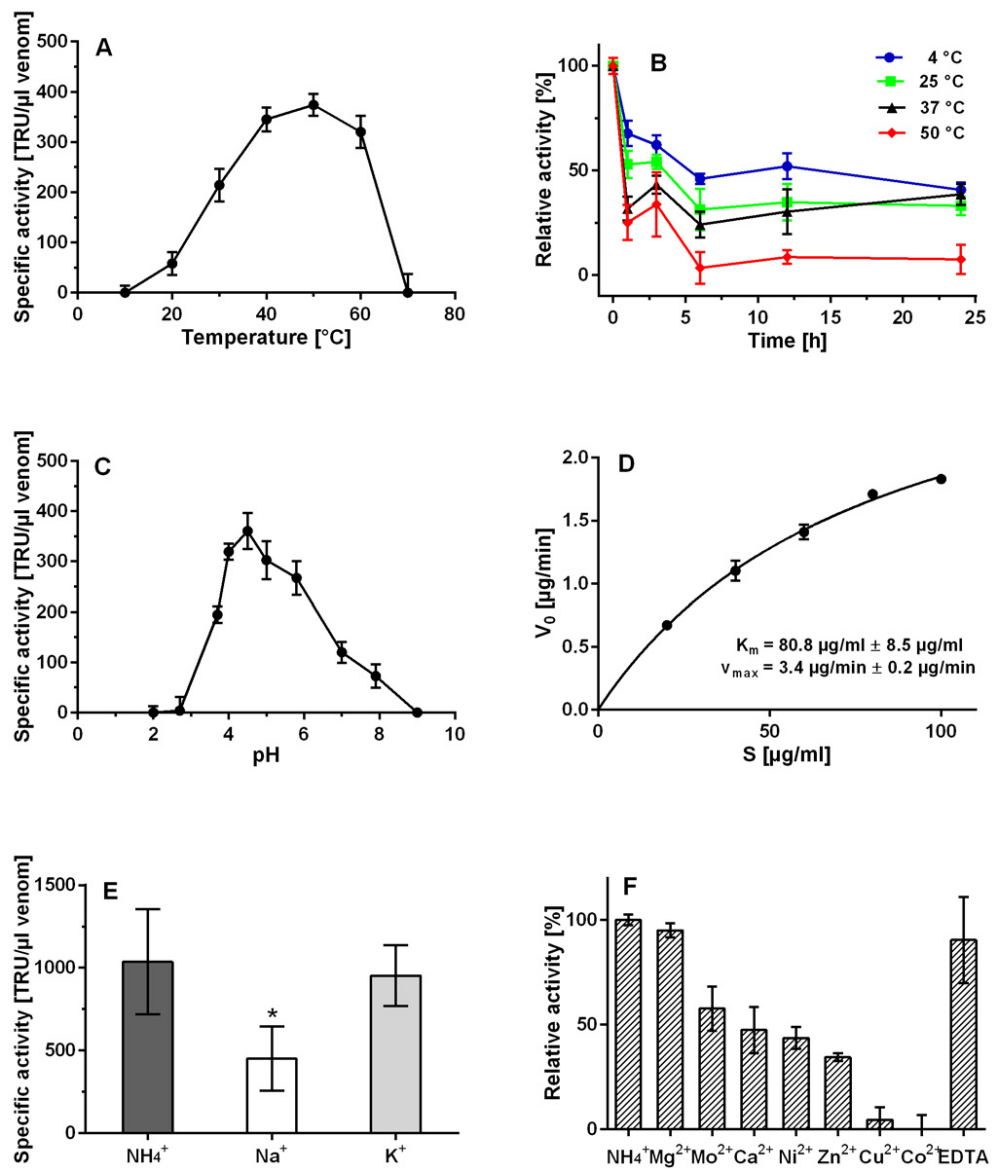
Enzymatic characterization of native CsHyal. (A) Effect of temperature on CsHyal activity. (B) Thermostability of CsHyal measured at 4, 25, 37, and 50°C after 1, 3, 6, 12, and 24 h of incubation. (C) pH profile of CsHyal activity. (D) Determination of K_m_ and V_max_ using non-linear regression. (E) Effect of monovalent salts in 0.2 M NH_4_^+^-buffer containing 0.2 M K^+^ or Na^+^, respectively. Activity decreased in Na^+^ compared to NH_4_^+^ (* significance, p = 0.007). (F) Influence of divalent cations and EDTA on CsHyal activity.

**Fig 7:**
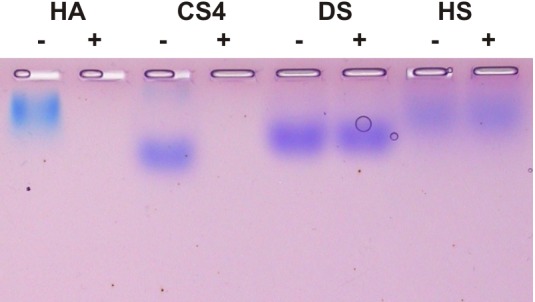
Substrate specificity of native CsHyal. HA, CS4, HS, and DS were incubated with 0.1 μl venom at 40°C overnight and separated on a 1% agarose gel at 50 mA for 3h. Degradation was revealed by sequential toluidine blue and Stains-All staining. Lanes labeled with + represent incubation in the presence of venom and those with - in the absence of venom.

*C*. *salei* venom showed maximal HA degradation activity of 280 TRU/μl venom under standard assay conditions (0.2 M NaAc, 0.15 M NaCl, pH 6, 37°C). CsHyal is optimally active at temperatures between 40–60°C (Fig 6A). Relative activity decreased within 1 h at 4°C by 30% and again by 30% in the next 5 h at 4°C. After 1 h at 25 and 37°C only 30% to 50% of the initial activity was still detectable, but no major decrease was observed in the next 23 h. At 50°C relative activity decreased in the first 3h of incubation to 30% and further to 5% in the next 3 h (Fig 6B). High activity was detected in the pH range from pH 4.0 to 6.0 (Fig 6C). The *K*_m_ and *V*_max_ values under standard assay conditions were found to be 80.8 μg/ml ± 8.5 μg/ml and 3.4 μg/min ± 0.2 μg/min, respectively, determined by non-linear regression (Fig 6D). Activity decreased in Na^+^-buffer to ~50% compared to NH_4_^+^-buffer (p = 0.007), whereas K^+^ showed no significant reduction of CsHyal activity (Fig 6E). The influence of different divalent metal cations (5 mM) on CsHyal activity was also investigated. Out of the seven metal salts tested, only the addition of Mg^2+^ did not affect the activity of CsHyal, all other tested divalent cations either reduced or fully inhibited CsHyal activity. It was not possible to measure the effect of Fe^2+^ and Mn^2+^, as both ions precipitated after addition of the stop solution. Addition of 5 mM EDTA did not affect CsHyal activity (Fig 6F). *C*. *salei* venom was able to degrade the GAGs HA and CS4 completely, whereas DS and HS were not affected (Fig 7).

### GAG degradation, carbohydrate analysis and TLC

**Fig 8:**
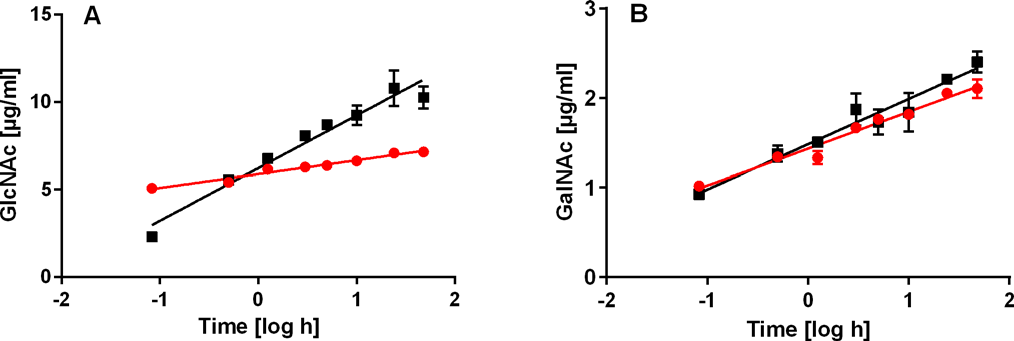
Time dependent production of terminal GlcNAc or GalNAc residues at the reducing end through *C*. *salei* venom and bovHyal. (A) Incubation of HA with *C*. *salei* venom (red circles) or bovHyal (black squares) resulted in a time dependent release of reducing GlcNAc as indirect measurement of Hyal activity. (B) Incubation of CS4 with *C*. *salei* venom (red circles) or bovHyal (black squares) resulted in a time dependent release of reducing GalNAc as indirect measurement of Hyal activity.

Degradation of HA or CS4 by *C*. *salei* venom and bovHyal resulted in oligosaccharides of different sizes containing always terminal GlcNAc or GalNAc at the reducing end. These results highlight CsHyal as an endo-β-N-acetyl-hexosaminidase hydrolase, comparable to bovHyal, as shown by the time dependent release of GlcNAc or GalNAc during the degradation of HA or CS4 (Fig 8A and 8B). The enzymatic activity of CsHyal is characterized by a nearly 2.2 times higher production of GlcNAc (5.08 μg/ml) within the first five min of incubation in contrast to bovHyal with 2.3 μg/ml. However, after this fast initial GlcNAc release by CsHyal, the HA degradation rate (CsHyal: 0.790 μg/(ml x h) ± 0.056 μg/(ml x h); bovHyal: 3.01 μg/(ml x h) ± 0.195 μg/(ml x h)) is significantly (F = 119.582; DFn = 1; DFd = 28; p < 0.0001) slower than the one of bovHyal resulting in a 1.4 fold lower total release of GlcNAc after 48 h (bovHyal: 10.3 μg/(ml x h); CsHyal: 7.1 μg/(ml x h)) (Fig 8A). Interestingly, the enzymatic activities of CsHyal and bovHyal, concerning CS4 degradation, exhibit no big difference of CS4 degradation rates (F = 4.285; DFn = 1; DFd = 28; p = 0.048) (CsHyal: 0.411 μg/(ml x h) ± 0.023 μg/(ml x h); bovHyal: 0.505 μg/(ml x h)± 0.0396 μg/(ml x h)) (Fig 8B). After the first five min, CsHyal degrades CS4 nearly five times less, when compared with HA degradation. Additionally, after 48 h of incubation, CS4 is 3.4 and 4.3 times less degraded than HA by CsHyal and bovHyal, respectively.

**Fig 9:**
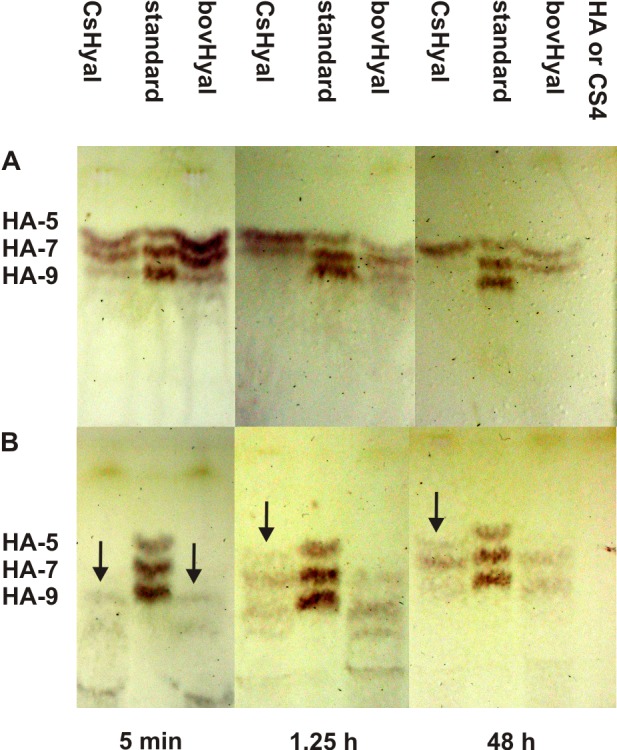
TLC of time dependent degradation products of HA and CS4 through hydrolysis with *C*. *salei* venom or bovHyal. (A) HA was incubated with *C*. *salei* venom or bovHyal for 5 min, 1.25 h and 48 h. (B) CS4 was incubated with *C*. *salei* venom or bovHyal for 5 min, 1.25 h and, 48 h as described under materials and methods. Standards were different HA-oligomers with an additional GlcNAc at the non-reducing end: HA-5, HA-7 and, HA-9. HA or CS4 without *C*. *salei* venom/bovHyal were applied as control.

For the first time, spider Hyal degradation products of HA and CS4 have been identified by TLC. Surprisingly, CsHyal degrades HA after 5 min mainly to HA-tetramers, HA-hexamers and to a lower extent to HA-octamers. After 1.25 h HA-octamers are not detectable anymore and after 48 h of incubation only HA-tetramers are detected, obviously the end products of HA degradation by CsHyal. BovHyal degrades HA mainly to HA-tetramers, HA-hexamers and HA-octamers as shown after 5 min and 1.25 h of incubation. As end products after 48 h, HA-tetramers and HA-hexamers are obtained (Fig 9A).

Degradation of CS4 by CsHyal resulted in three faint bands after 5 min, one corresponding to CS4-octamers and the two other bands to higher CS4-oligomers. After 1.25 h, mainly CS4-hexamers and CS4-octamers have been identified, but additionally traces of CS4-tetramers and higher CS4-oligomers are visible. Long time incubation (48 h) resulted in a mixture of CS4-tetramers, CS4-hexamers and CS4-octamers as after 1.25 h, but the CS4-hexamers is the strongest band visible. In contrast, after 1.25 h bovHyal degrades CS4 to CS4-octamers and in a lower extent to CS4-hexamers, as well as higher CS4-oligomers. End products of CS4 cleavage after 48 h of incubation are mainly CS4-hexamers and CS4-octamers (Fig 9B).

### Expression of recombinant CsHyal

**Fig 10:**
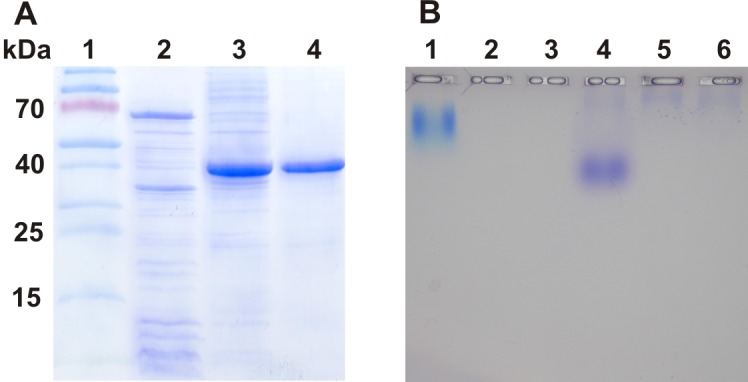
Purification of rCsHyal and substrate specificity of purified rCsHyal. (A) Solubility of expressed rCsHyal and the purity of separated and refolded rCsHyal were analyzed by SDS PAGE (12%). rCsHyal was only found in the insoluble fraction and was therefore purified under denaturing conditions. Lanes: 1, PageRuler Prestained Protein Ladder (Thermo Scientific); 2, soluble fraction after cell lysis; 3, insoluble fraction after cell lysis; 4, rCsHyal after purification and refolding. (B) HA (lane 1, 2 and 3) and CS4 (lane 4, 5 and 6) were incubated with approximately 1.52 TRU (lane 2 and 5) and 0.76 TRU (lane 3 and 6) rCsHyal at 40°C overnight and separated on a 1% agarose gel at 50 mA for 3h. Degradation was revealed by sequential toluidine blue and Stains-All staining.

After expression of rCsHyal in a pET28a (+) vector and in BL21(DE3)pLysS cells the recombinant protein was only detected in the insoluble part of the cell lysate (Fig 10A). Extraction of rCsHyal from inclusion bodies was achieved with 8 M urea or 6 M guanidium hydrochloride, but only affinity chromatography in guanidium hydrochloride led to pure rCsHyal (Fig 10A). Purification of rCsHyal under denaturing conditions yielded 4.3 mg pure rCsHyal per liter LB-medium. In contrast to affinity purification refolding did only work in urea and not in guanidium hydrochloride and therefore rCsHyal was dialyzed against 8 M urea after purification in 6 M guanidium hydrochloride. Several refolding methods were tested, but only with the protocol described in [37] HA degradation activity was detected (data not shown). After refolding rCsHyal exhibited a specific activity of 986.4 TRU/mg.

Due to the high content of Na^+^ (147 mM) and K^+^ (4.5 mM) in the refolding buffer, rCsHyal was further desalted on a PD-10 column against bidistilled water, resulting in a reduced specific activity of 290.3 TRU/mg. Dialysis of the refolded rCsHyal against bidistilled water did not result in any detectable HA degradation activity. Despite of the missing glycosylations of rCsHyal and a 10.7-fold reduced specific activity compared to pure native CsHyal, the enzyme was still able to degrade HA as well as CS4 (Fig 10B) and therefore useful for bioassays.

### Bioassays

**Fig 11:**
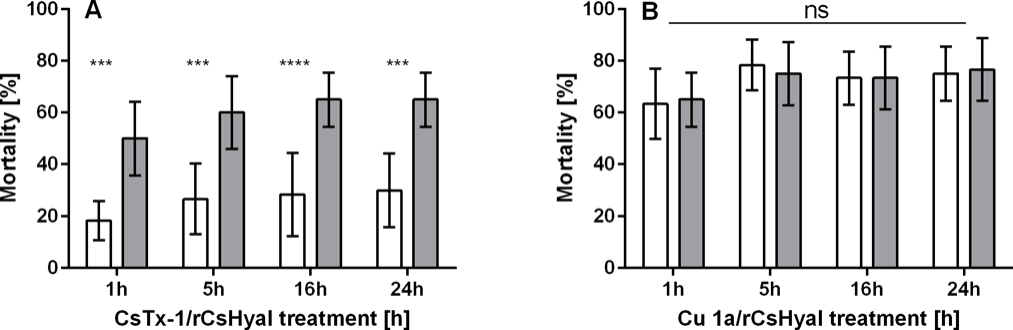
Bioassays with *D*. *melanogaster*. Not shown are control injections of only buffer or rCsHyal (0.42 TRU/mg fly) which were completely non-toxic. (A) The neurotoxin CsTx-1 (0.40 pmol/mg fly) was injected alone (open bars) or in combination with rCsHyal (0.42 TRU/mg fly) (gray bars) and the total paralysis/mortality was determined after 1, 5, 16, and 24h. **** corresponds to p < 0.0001 and *** corresponds to p < 0.001. (B) The cytolytic acting Cu 1a (7.45 pmol/mg fly) was injected alone (open bars) or in combination with rCsHyal (0.42 TRU/mg fly) (gray bars) and the total paralysis/mortality was determined after 1, 5, 16, and 24h. ns means not significant.

Injection of 0.42 TRU rCsHyal/mg fly showed no effect on *Drosophila* flies when compared with the control group. Both groups recovered within 5 minutes after injection. Injection of 0.40 pmol CsTx-1/mg fly caused a complete paralysis/mortality of 18% after 1h and of 30% after 24h (Fig 11A, CsTx-1). Coinjection of 0.40 pmol CsTx-1/mg fly with 0.42 TRU rCsHyal/mg fly increased the mortality after 24h 2.2-fold from 30 to 65% (p < 0.001), exhibiting a synergistic effect of rCsHyal (Fig 11A, CsTx-1/ rCsHyal). The observed complete paralysis/mortality of the flies between 1h, 5h, 16h, and 24h after injection of CsTx-1 alone or in combination with 0.42 TRU rCsHyal/mg fly showed no statistically significant difference, indicating fast activity. Injection of 7.45 pmol Cu 1a/mg fly caused a complete paralysis/mortality of 63% after 1h and of 75% after 24h (Fig 11B, Cu 1a). Coinjection of 7.45 pmol Cu 1a /mg fly with 0.42 TRU rCsHyal/mg fly resulted in a complete paralysis/mortality of 77%, showing no synergistic effect of rCsHyal on the cytolytic activity of Cu 1a (Fig 11B, Cu 1a/rCsHyal). The observed paralysis/mortality of the flies between 1h, 5h, 16h, and 24h after injection of Cu 1a alone or in combination with 0.42 TRU rCsHyal/mg fly showed no statistically significant difference.

### Evolution of the Hyal-like enzyme on spider family level

Venom of 39 spider species of 21 different families was incubated with HA, CS4, DS, and HS to examine occurrence and substrate specificity of spider venom Hyal-like enzymes (Table 3, S3 Fig). From four tested species belonging to the suborder Mygalomorphae, only *Atypus piceus* (Atypidae), showed no hydrolytic activity towards any of the tested GAGs. *Linothele megatheloides* (Dipluridae) and *Macrothele calpeiana* (Hexathelidae) degraded HA completely and CS4 partially, whereas *Sericopelma rubronitens* venom (Theraphosidae) showed complete degradation of HA and CS4. All other tested spider venoms were from species belonging to the suborder Araneomorphae. Venom from *Filistata insidiatrix* (Filistatidae), *Segestria florentina* (Segestriidae), *Latrodectus tredecimguttatus*, and *Steatoda paykulliana* (Theridiidae) was able to degrade HA as well as CS4 completely. The venom from *Meta menardi* (Tetragnathidae), *Callobius claustrarius* (Amaurobiidae), and *Uroctea durandi* (Oecobiidae) degraded HA completely and CS4 partially. No GAG degradation was observed for the venom of *Eresus walckenaerius*, *Stegodyphus lineatus* (Eresidae), *Nephila pilipes* (Nephilidae), *Araneus angulatus*, *Araneus diadematus*, *Argiope bruennichi*, and *Larinioides sclopetarius* (Araneidae). Complete HA and CS4 hydrolysis was observed for the following species belonging to different families of the retrolateral tibial apophysis (RTA) clade: *Tegenaria atrica*, *Agelena labyrinthica*, *Polybetes pythagoricus*, *Isopeda villosa*, *Eusparassus dufouri*, *Drassodes lapidosus*, *Zoropsis spinimana*, *Oxyopes* sp., *Dolomedes okefinokensis*, *Pisaura mirabilis*, *Alopecosa fabrilis*, *Alopecosa marikovskyi*, *Geolycosa vultuosa*, *Hogna radiata*, *Lycosa hispanica*, *Lycosa praegrandis*, *Ancylometes rufus*, *C*. *salei*, *Viridasius fasciatus*, *Phoneutria fera*, *and Phoneutria reidyi*. None of the tested venoms was able to degrade DS or HS (Table 3, S3 Fig).

**Table 3:** Substrate specificity of spider venom Hyals. Degradation (+), partial degradation (+/-) or no degradation (-) of the examined substrates (HA, CS4, DS, HS) after incubation with spider venom. The degradation process was recorded after agarose gel electrophoresis and subsequent staining with toluidine blue and Stains-All.

Family	Species	Degraded GAGs			
		HA	CS4	DS	HS
**Mygalomorphae**					
Atypidae	*Atypus piceus*	-	-	-	-
Dipluridae	*Linothele megatheloides*	+	+/-	-	-
Hexathelidae	*Macrothele calpeiana*	+	+/-	-	-
Theraphosidae	*Sericopelma rubronitens*	+	+	-	-
**Araneomorphae**					
Filistatidae	*Filistata insidiatrix*	+	+	-	-
Segestriidae	*Segestria florentina*	+	+	-	-
Eresidae	*Eresus walckenaerius*	-	-	-	-
	*Stegodyphus lineatus*	-	-	-	-
Oecobiidae	*Uroctea durandi*	+	+/-	-	-
Theridiidae	*Latrodectus tredecimguttatus*	+	+	-	-
	*Steatoda paykulliana*	+	+	-	-
Tetragnathidae	*Meta menardi*	+	+/-	-	-
Nephilidae	*Nephila pilipes*	-	-	-	-
Araneidae	*Araneus angulatus*	-	-	-	-
	*Araneus diadematus*	-	-	-	-
	*Argiope bruennichi*	-	-	-	-
	*Larinioides sclopetarius*	-	-	-	-
**RTA-clade** *					
Amaurobiidae	*Callobius claustrarius*	+	+/-	-	-
Agelenidae	*Tegenaria atrica*	+	+	-	-
	*Agelena labyrinthica*	+	+	-	-
Sparassidae	*Eusparassus dufouri*	+	+	-	-
	*Isopeda villosa*	+	+	-	-
	*Polybetes pythagoricus*	+	+	-	-
Gnaphosidae	*Drassodes lapidosus*	+	+	-	-
Zoropsidae	*Zoropsis spinimana*	+	+	-	-
Pisauridae	*Dolomedes okefinokensis*	+	+	-	-
	*Pisaura mirabilis*	+	+	-	-
Oxyopidae	*Oxyopes* sp.	+	+	-	-
Lycosidae	*Alopecosa fabrilis*	+	+	-	-
	*Alopecosa marikovskyi*	+	+	-	-
	*Geolycosa vultuosa*	+	+	-	-
	*Hogna radiata*	+	+	-	-
	*Lycosa hispanica*	+	+	-	-
	*Lycosa praegrandis*	+	+	-	-
Ctenidae	*Ancylometes rufus*	+	+	-	-
	*Cupiennius salei*	+	+	-	-
	*Phoneutria fera*	+	+	-	-
	*Phoneutria reidyi*	+	+	-	-
	*Viridasius fasciatus*	+	+	-	-

*RTA-clade (araneomorph spider families exhibiting a **R**etrolateral **T**ibial **A**pophysis)

**Fig 12:**
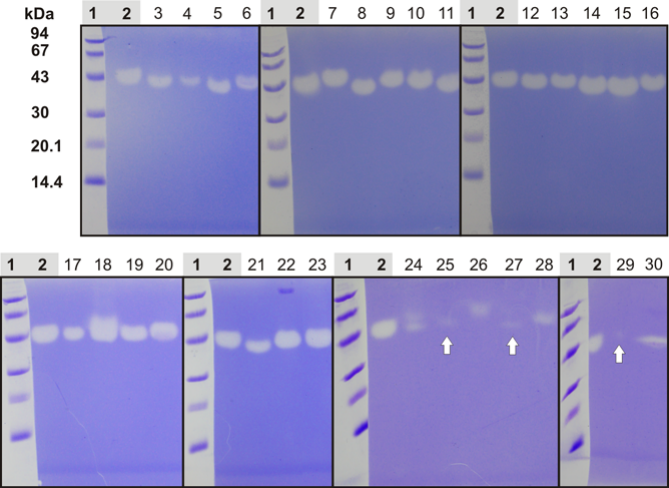
Hyaluronidase activity of different spider venoms. Different spider venoms were separated in a 10% SDS-PAGE which was copolymerized with HA as substrate for Hyals. The dilution of different venoms is given in brackets. Lanes: 1, low molecular mass standard (GE Healthcare); 2, *C*. *salei* (1:100); 3, *Phoneutria fera* (1:20); 4, *Phoneutria reidyi* (1:10); 5, *Eusparassus dufouri* (1:10); 6, *Uroctea durandi* (1:10); 7, *Ancylometes rufus* (1:100); 8, *Viridasius fasciatus* (1:50); 9, *Hogna radiata* (1:100); 10, *Alopecosa fabrilis* (1:100); 11, *Lycosa praegrandis* (1:100); 12, *Dolomedes okefinokensis* (1:100); 13, *Zoropsis spinimana* (1:100); 14, *Polybetes pythagoricus* (1:100); 15, *Isopeda villosa* (1:100); 16, *Lycosa hispanica* (1:100); 17, *Geolycosa vultuosa* (1:10); 18, *Pisaura mirabilis* (1:20); 19, *Macrothele calpeiana* (1:20); 20, *Meta menardi* (1:10); 21, *Latrodectus tredecimguttatus* (1:100); 22, *Sericopelma rubronitens* (1:100); 23, *Drassodes lapidosus* (1:10); 24, *Steatoda paykulliana* (1:5); 25, *Alopecosa marikovskyi* (1:5); 26, *Linothele megatheloides* (1:5); 27, *Oxyopes* sp. (1:5); 28, *Segestria florentina* (1:5); 29, *Filistata insidiatrix* (1:5); and 30, *Tegenaria atrica* (1:20).

Zymograms of representative venoms exhibit comparable molecular masses around 43 kDa (Fig 12), corresponding to the determined molecular mass of CsHyal. However, spider Hyals from *Ancylometes rufus* (lane 7), *Hogna radiata* (lane 9), *Alopecosa fabrilis* (lane 10), and *Linothele megatheloides* (lane 26) appear apparently with a slightly higher molecular mass compared to *C*. *salei* and in the opposite direction, venoms from *Eusparassus dufouri* (lane 5), *Polybetes pythagoricus* (lane 14), *Isopeda villosa* (lane15), and *Latrodectus tredecimguttatus* (lane 21) show a slightly lower molecular mass.

## Discussion

### Purification of native CsHyal

HA degradation assays with crude *C*. *salei* venom showed Hyal activity and a Hyal-like enzyme with a specific activity of 3,115 TRU/mg was purified applying a three step purification protocol. The loss of activity during RP-HPLC is most likely explained by denaturation of CsHyal by the high amount of acetonitrile needed to elute CsHyal from the column and the low pH of the RP-HPLC solvents. CsHyal was detected in the venom at a concentration of 1.3–9.3 μM and is up to 1000 times less abundant than the *C*. *salei* main neurotoxins CsTx-1 (1.4–3.3 mM) and CsTx-9 (0.2–1.1 mM) [50]. The low concentration might be explained by the fact that neurotoxins act stoichiometrically by binding to ion channels in electro-excitable cells [29], whereas CsHyal acts as an enzyme. This low abundance is the main reason for needing more than 5 ml of venom (collected over 5 years by milking of ~600 spiders), to enable biochemical characterization of native CsHyal.

### cDNA structure of CsHyal

The cDNA structure of CsHyal encodes for a short signal peptide comprising 16 amino acid residues followed by the mature protein and a stop signal. Actually, three further complete cDNA sequences of spider venom Hyals are available, but only for *Loxosceles intermedia* a 19 residue long signal peptide was assigned [16]. In the case of *Acanthoscurria geniculata* [51] and *Brachypelma vagans* [J9XYC6], both belonging to mygalomorph spiders, the reported N-terminal fragments are composed of at least 37 and 41 amino acid residues, respectively, and a cleavage site for a signal peptidase could not be assigned using SignalP 4.1 Server. However, the occurrence of a propeptide following the signal peptide was assumed not only for arachnid Hyals, but reported for insect and mollusks Hyals. In contrast to *C*. *salei* and *Loxosceles intermedia* Hyals, the signal peptide is followed by a propeptide in cone snail *Conus consors*, both 18 amino acid residues long [52], and honey bee Hyals (signal peptide 28 and propeptide 5 amino acid residues) [Q08169]. No information is available regarding the relevance of these propeptides.

### Structural characterization of native CsHyal

So far only few venom Hyals were analyzed on the protein level and most available sequences were extracted from cDNA data. It was shown that venom Hyals of yellow jacket *Vespula vulgaris*, honey bee *Apis mellifera*, and cone snail *Conus consors* are N-glycosylated with various glycoforms, while no O-glycosylation sites were identified [52–54]. Hyals in *Vespula vulgaris* and *Apis mellifera* venom are major allergens and N-linked glycans seem to be responsible for the cross-reactivity of these venoms [53]. Up to now, no data about glycosylations in arachnids are available.

Purified native CsHyal was used for structural characterization at the protein level by a combination of mass spectrometry, N-terminal sequence analysis, and amino acid analysis, all of them confirming the obtained cDNA sequence. Further MS/MS experiments revealed the glycosylation structure of CsHyal. As in *Vespula vulgaris* and *Apis mellifera* a heterogeneous mixture of paucimannosidic N-glycans with various degrees of fucosylation was identified in CsHyal, whereas no fucosylated glycans were identified in *Conus consors* Hyal [52–54].

Spider and scorpion bites can lead to dermonecrosis and allergic reactions causing unresolved clinical problems. While for spider bites, dermonecrosis is usually attributed to sphingomyelinase D and thus to *Loxosceles* and *Sicarius* bites (both Sicariidae), it was shown that Hyals may contribute to these effects and anti-Hyal serum against *Tityus serrulatus* Hyal neutralized lethal effects of this venom in mice [55, 56]. As the N-glycans of CsHyal resemble the allergenic glycoforms of *Vespula vulgaris* and *Apis mellifera*, it is possible that they might contribute to allergic reactions of spider venoms, but data are lacking.

### Structure modeling

The structural comparison between crystal structures of human Hyal-1, honey bee venom Hyal, and a model of CsHyal clearly showed that these enzymes have the same overall structure and share very similar active sites. Closer inspection of the active site residues revealed that CsHyal shows higher structural alignment with honey bee venom Hyal than human Hyal-1 indicating that the catalytic activity of CsHyal is closer to honey bee venom Hyal (Fig 5E). Amino acid residues important for the catalytic center of the enzyme, such as Y75, D129, E131, Y202, Y247 (referring to human Hyal-1 nomenclature), are strictly conserved in all aligned sequences (S4 Fig). The putative disulfide bridge between Cys171 and Cys213 was identified in spider and scorpion venom Hyal sequences and might be characteristic for arachnid Hyals [30]. However, this putative disulfide bridge seems to be already present in a *C*. *elegans* Hyal-like enzyme (Cys162 and Cys259 or Cys262) [57].

Interestingly, CsHyal exhibits ~ 50% identity with other spider venom Hyals (*L*. *intermedia* and *B*. *vagans*) and ~ 42% identity with scorpion venom Hyals (*M*. *martensii* and *T*. *serrulatus*), which seems to reflect their phylogenetic proximity. Reduced identities (34.4–36.9%) are obtained when the CsHyal sequence is compared with insects, which do not possess the C-terminal EGF-like structure. Comparison with vertebrate Hyals, containing a C-terminal EGF-like structure, results in identities between 30.2–36.4% (S4 Fig).

All available spider venom Hyal sequences exhibit a C-terminal EGF-like domain, which is absent in honey bee Hyal, but present in *Conus consors* Hyal [52] (S4 Fig). So far, the function of the EGF-like domain in Hyals is unclear. The assumed EGF-like domain may mediate protein-protein interactions as proposed for vertebrate Hyals [48]. This domain and the glycosylation at N360 may play, beside the HA/CS4 cleavage activity of CsHyal, a second so far unknown role concerning spreading of toxic compounds.

### Enzymatic activity characterization

CsHyal shows biochemical characteristics similar to other venom Hyals. The enzyme showed HA degradation between 20 and 60°C, with a temperature optimum around 50°C. Loss of activity was observed above 60°C. Even though highest activity was found at 50°C, the enzyme is not very stable at this temperature. Over 75% of its activity is lost after 1h at 50°C and over 90% after 6h of incubation. At 25°C and 37°C, the enzyme lost 50–70% of its activity during the first hour of incubation, but retains the remaining activity for the next 23 h. The high loss of activity during the first hour might be explained by the dilution of the venom required for measurements, which changes the environment of the enzyme. With a pH optimum of 4.5, CsHyal belongs to the acid-active class of Hyals [15], such as scorpion venom Hyals of *Palamneus gravimanus* and *Buthus martensii* and the spider venom Hyal of *Vitalius dubius* [4, 8, 58]. Spider Hyal of *Hippasa partita*, in contrast, is optimally active at pH 6 [21]. CsHyal was more active in 0.2 M K^+^ than in 0.2 M Na^+^. This is in agreement with *C*. *salei* venom ion concentrations, which are 9 mM Na^+^ and 215 mM K^+^ [29]. Addition of EDTA to the assay buffer did not affect the activity of the enzyme and none of the tested divalent cations increased the degradation activity. This indicates that no endogenous divalent metal ion is required for optimal catalysis. Addition of Mg^2+^ did not show any effect, while all other metal salts (Ca^2+^, Cu^2+^, Co^2+^, Mo^2+^, Ni^2+^, and Zn^2+^) reduced the activity of CsHyal. It has been reported that the influence of divalent cations is rather linked to the substrate than the enzyme, as metal salts can bind to HA and influence its conformation [59]. The *K*_m_ of 80.8 μg/ml is in the same range as scorpion venom Hyals of *Palamneus gravimanus* (47.61 μg/ml) and *Tityus serrulatus* (69.7 μg/ml) [8, 9]. This indicates a comparatively high affinity of HA for the binding cleft, in contrast to the spider venom Hyals of *Loxosceles intermedia* (709 μg/ml) and *Vitalius dubius* (677 μg/ml) [13, 58].

### Hyal substrate specificity and degradation end products

Comparable to bovHyal, CsHyal hydrolyses the β-1,4 glycosidic bond between GlcNAc (HA) or GalNAc (CS4) and GlcA as verified by the determination of increasing amounts of terminal GlcNAc or GalNAc at the reducing end for both substrates (Fig 8A and 8B). Both enzymes degrade HA about two times more efficiently than CS4. Interestingly, CsHyal is within the first 5 min more active than bovHyal, but overall bovHyal shows a higher amount of HA degradation than CsHyal. This points to a specialization of bovHyal, mainly a compound of sperm, to degrade hyaluronan in the corona radiata of eggs [60]. CsHyal shows no higher efficiency towards CS4 hydrolysis though this is the prevailing substrate in the spiders’ nutritional environment. However, CS4 and HA hydrolysis may not be the only function of CsHyal. Comparable to sperm Hyal PH-20, which besides its HA hydrolyzing activity, is involved in intracellular signaling [61], also CsHyal may have additional still unknown functions, which may help destroying the tissue around the injection location for a better susceptibility of the toxic compounds from spider venom as generally hypothesized for Hyals [20].

The analysis of the degradation products of HA by CsHyal or bovHyal reveals that there might be differences in the catalytic activity of both enzymes. Five min of incubation yield HA-tetramers, HA-hexamers and HA-octamers for both enzymes. However, after 75 min CsHyal activity results mainly in HA-tetramers and HA-hexamers, whereas bovHyal activity results mainly in HA-hexamers and less in HA-tetramers and HA-octamers. After 48 h of incubation, the CsHyal end products are characterized only by HA-tetramers, also reported for scorpion Hyal final degradation products [44]. In contrast to bovHyal, where both HA-tetramers and HA-hexamers have been identified, which is in accordance with the current knowledge [62].

The hydrolysis of CS4 after 5 minutes is comparable for both enzymes and CS4-octamers appear as faint bands in TLC. After 75 min, CsHyal activity resulted in a set of octameric, hexameric and tetrameric degradation products and after 48 h, besides tetramers and octamers, mainly hexamers are visible in contrast to bovHyal (Fig 9B). Generally, the end products of CS4 and HA cleavage by CsHyal are one disaccharide unit smaller than the end products generated by bovHyal. Such results may point to differences in the catalytic activity of both enzymes and may be a specific adaption towards CS4 or HA degradation. Importantly, these small end products may act by influencing edema, inflammation, and modulating in vertebrates a diversity of immune responses [63]. Furthermore, oligomers of HA activate nitric oxide synthase and as a result NO production has been shown for different cell types [64].

### Recombinant protein expression

In consequence of limited *C*. *salei* venom and the low venom concentration of CsHyal (1.3–9.3 μM), expression of rCsHyal was essential for performing further bioassays. As in the expression of *Loxosceles intermedia* Hyal [16], rCsHyal was only found in inclusion bodies and had to be extracted under denaturing conditions. As CsHyal exhibits several disulfide bridges (Fig 5F) and purification was done under denaturing conditions, the enzyme had to be reduced and refolded. Hofinger et al. [64] tested several conditions to refold human Hyal-1 and the refolding condition, with which they found the highest enzymatic activity, did also result in active rCsHyal. The specific activity of pure rCsHyal (986.4 TRU/mg) after refolding was much lower than the one of the purified native CsHyal (3,115.0 TRU/mg) and desalting of rCsHyal lowered the specific activity further to 290.304 TRU/mg. Notwithstanding of the low specific activity of desalted rCsHyal, recombinant protein expression allowed purification of enough active rCsHyal for bioassays. For two vertebrate Hyals, human Hyal-1 [65] and PH-20 [66], it could be shown that deglycosylation reduced the enzyme activity, which may explain the low activity of rCsHyal. Zhang et al. [65] showed that deglycosylation at N350 of human Hyal1, adjacent to the EGF-like domain, is mainly responsible for lacking complete activity and that glycosylation at position N216 may facilitate folding and maturation of the enzyme.

### Bioassays

The function of spider venom Hyal-like enzymes as ‘spreading factor’ and its toxic effects on vertebrates have been investigated [16, 67, 68], but only one publication concerning toxic effects on insects is available. In 1973, it was reported that a size exclusion chromatography fraction from the venom of the spider *Aphonopelma hentzi* [11] exhibited Hyal activity, which showed little or no toxicity towards German cockroaches. Only eight cockroaches were used for testing the Hyal containing fraction and eight cockroaches as the control group. A clear statement concerning the toxic effects was not possible because also in the control group one insect died.

Due to the low concentration of native CsHyal in the venom (~ 0.06–0.43 μg/μl), recombinant rCsHyal was used for all bioassays. To obtain rCsHyal free of Na^+^ and K^+^ ions for these assays, refolded rCsHyal was desalted and reduced the specific activity of rCsHyal by a factor of 3.4. As desalted rCsHyal was still active and exhibited degradation of HA and CS4 (Fig 7B), recombinant enzyme was used for bioassays in the same activity range. Injection of 0.42 TRU/0.05 μl rCsHyal into *Drosophila* flies showed no effect and therefore rCsHyal was completely non-toxic at the tested concentration. Furthermore, the activity is about 13 times less when compared with physiological activity of CsHyal (5.3 TRU/0.05 μl venom). The high protein content and viscosity of rCsHyal prevented a higher dose injection into *Drosophila* flies.

Coinjection of a non-toxic dose of rCsHyal with the main *C*. *salei* venom neurotoxin CsTx-1 led to a statistically significant (p < 0.001) increase of mortality (2.3-fold) compared to the mortality of the neurotoxin injected alone. This synergism highlights the function of CsHyal in the venom as spreading factor. Furthermore, the spreading effect of rCsHyal seems to occur within a short timeframe, because no significant increase of complete paralysis/mortality was detected between 1h and 24h after coinjection of CsTx-1 and CsHyal.

However, coinjection of rCsHyal and the membranolytic acting peptide Cu 1a showed no synergistic effect. The molecular basics of this interesting effect are not yet understood and have to be investigated further. Nevertheless, it might be possible that Cu 1a directly binds to a wide variety of negatively charged membrane types within the prey or in the case of defending, within the predator [69], which may not be directly enclosed by connective tissue and therefore a synergistic effect of rCsHyal on the activity of Cu 1a was not observed. As reported earlier, coinjection of Cu 1a with CsTx-1 synergistically increases the toxic activity of CsTx-1 [50]. It is tempting to speculate that both, CsHyal and Cu 1a may act as spreading factors aiming at different targets, GAGs and membranes, and therefore enhance the activity of neurotoxic venom compounds.

Overall, our data indicate the spreading effect of spider venom Hyal-like enzymes in insects, which probably represents the role for which the enzyme has been recruited into the venom of spiders [70].

### Distribution and substrate specificity of spider venom Hyal-like enzymes

As only a few spider families were tested for venom Hyal activity so far, we investigated the extent of Hyal occurrence in spider phylogeny (Table 3, S3 Fig). To investigate substrate specificity of different spider venom Hyal-like enzymes, four different GAGs (HA, CS4, DS, HS) were incubated with venom overnight, separated by agarose gel electrophoresis, and sequentially stained by toluidine blue and Stains-All. Investigation of the substrate specificity of venom Hyals was not performed with the conventional turbidometric assay or zymography, because, in contrast to HA, the GAGs CS4, DS, and HS exhibit lower molecular masses, which might cause misinterpretation of the obtained data as reported by Honda et al. [26].

We found a wide distribution of venom Hyal activity in 17 out of 21 tested families throughout the phylogenetic tree of spiders, indicating the importance of this enzyme as venom component. The zymograms of different representative Hyal containing venoms show only small molecular mass differences, which may reflect various glycosylation pattern of the enzyme or refer to amino acid sequence differences.

However, for some species we did not find any hydrolytic activity towards the tested GAGs. The venom of *Atypus piceus*, a representative of the oldest spider family (Atypidae) in our investigation, showed no Hyal activity. It has functional venom glands and this result leads to the fascinating question, whether atypids did not yet have a venom Hyal (then it would be an autapomorphy of all higher spiders) or if it lost it already (then Hyal would be ancient heritage of spiders). The non-possession of Hyal could be explained by the peculiar prey catching habit of atypids within a silken tube where prey items are pulled inside the tube and can no more escape. Also both eresid species showed no Hyal activity in their venom, which is consistent with a previous study reported by Sanggaard et al. [51], where neither a Hyal among the venom proteins nor a Hyal gene were found in the velvet spider *Stegodyphus mimosarum* [51]. A reason for their Hyal loss is not obvious. Solitarily living *Eresus* and social *Stegodyphus* species have very different hunting strategies and web types. They are both cribellate spiders but other cribellate spiders (Filistatidae and Amaurobiidae) show normal Hyal activity. Also Oecobiidae, the sister family of Eresidae, shows normal Hyal activity. As a third group, all five species of the sister families Araneidae and Nephilidae showed no Hyal activity, in accordance with the study by Duan et al. [71], where no Hyal gene candidate was identified in the cDNA library of the venom gland of the araneid spider *Araneus ventricosus*. It is likely that these spiders lost Hyal-like enzymes in the venom during evolution, perhaps due to their specific hunting strategy and/or venom composition. Araneids and nephilids are orb web-building spiders with one of the most-sophisticated prey wrapping behaviors among spiders. Compared to free hunting wandering spiders, orb web-building spiders may not need to rely on fast acting venom to subdue their prey, since prey items are wrapped immediately by the spider, prior to the venom bite, to avoid escape of the prey.

The here presented data provide good arguments for the hypothesis that the loss of Hyal activity in spider venoms is facilitated by alternative prey catching and wrapping methods. This would delimit Hyal loss in spider venoms to web-building spiders. As reason for such a loss we propose energetic efficiency, thus the possibility to save additional costs for Hyal synthesis. This idea is supported by the Uloboridae, the only spider family which reduced not only Hyal but also the total venom glands completely. They replaced them by the most intensive prey wrapping behavior ever detected in spiders.

The venom of the two mygalomorph species from the families Dipluridae and Hexathelidae showed complete degradation of HA and partial degradation of CS4. Also the venoms of *Callobius claustrarius* (Amaurobiidae) showed complete degradation of HA, but only partial degradation of CS4. Except for *C*. *claustrarius*, all tested species belonging to the RTA-clade had a venom Hyal-like enzyme that was able to degrade HA as well as CS4 completely. This correlates with the low abundance of Hyal in the venom of these spiders (only moderate dilution needed for zymograms), while all other spiders possess a highly abundant Hyal (dilution 1:100 needed).

All these species prey predominantly on arthropods. However, spiders of the families Agelenidae, Sparassidae, Pisauridae, Lycosidae, and Ctenidae have been reported to include occasionally small vertebrates into their diet as well [72–76]. Thus, those spiders may benefit by having a venom Hyal-like enzyme with broad substrate specificity, which is able to degrade the GAGs in invertebrates as well as in vertebrates. Some of the tested spider species have venom Hyal-like enzymes with an altered substrate specificity or loss of Hyal activity. Probably, these alterations resulted from mutations in the catalytic domain [55, 77], which led to changes in the substrate specificity [77] or resulted in loss of the Hyal activity [55, 77, 78].

Taking our results into account the question arises, what the main function of spider venom Hyals is and consequentially, the correct term for this enzyme in spider venom. As mentioned by Stern [1], ‘the term Hyal is somewhat of a misnomer’, because this diverse enzyme group identified in bacteria, invertebrates, and vertebrates is, with a few exceptions, able to depolymerize both, HA and Ch/CS [1, 20, 25]. Only *C*. *elegans* chondroitin hydrolase homologous to human Hyals showed hydrolysis preference for Ch over HA [28]. From an evolutionary point of view chondroitinases and their substrates Ch and CS seem to have appeared earlier than Hyals and HA [20, 22]. Therefore, it is tempting to speculate that spider venom Hyals developed from an ancestral chondroitinase and should be named chondroitinases due to the lack of HA in arthropods. However, spider venom Hyals exhibit hydrolytic activities towards HA and CS, so the name chondroitinase should be reserved to enzymes acting exclusively or preferentially on Ch/CS [28] and Hyal for enzymes that mainly cleave HA. One possibility would be to term spider venom Hyals, which degrade HA and CS, Hyal-like enzymes.

HA is only present in bacteria, vertebrates, and mollusks [22, 25, 79], whereas CS was identified in many invertebrates from coelenterates to plathelminthes, and nematodes up to arthropods, and from echinoderms to vertebrates. The occurrence of Ch was reported in *C*. *elegans* [25] and by quantitative disaccharide analysis, both Ch and CS were identified in *D*. *melanogaster* [80]. It is currently hypothesized that Ch is more ancient than HA and its likely precursor [20].

Although previous studies showed that Hyals in spider venom degrade HA at a much higher rate than CS4 [21, 58], CS4 might be the prime substrate for venom Hyal-like enzymes in spiders preying on insects and other arthropods, which is also important in defending against other arthropod predators. It is likely that the role of CS4 as substrate for spider venom Hyal-like enzymes has been underestimated in previous studies, since turbidimetric assays and zymography were used for the detection of Hyal activity. Honda et al. stated in 2012 [26] that these methods are only applicable to high molecular mass substrates such as HA (4–8’000 kDa) and it is therefore not possible to compare HA and CS4 degradation quantitatively using turbidimetric assays and zymography [81]. Hence, the rather small CS4 (5–50 kDa) [81] cannot be measured reliably with these methods. Using the highly sensitive method for measuring GAG degradation introduced by Kaneiwa et al. [57], Honda et al. [26] showed that human Hyals, Hyal-1 and SPAM1 degrade HA, Ch, and CS4 to a comparable extent. Both Hyals were thought to hydrolyze CS4 only at a limited rate. Thus, although termed Hyals, it appears that CS may be as important as substrate for spider venom Hyals as HA and consequently they should be named Hyal-like enzymes. This is supported by our results concerning the spreading activity of rCsHyal towards *Drosophila* flies in which Ch and CS, but no HA has been identified [76, 80]. Nevertheless, in some spider species also HA might act as substrate, since their defensive or predacious behavior is not only directed towards arthropods, but also species that contain HA [76] such as vertebrates.

### Conclusions

Our results suggest that besides HA, CS serves as substrate for most spider venom Hyal-like enzymes, which allows spider venom components to spread in vertebrate prey and predators exhibiting HA. Invertebrate prey and predators containing CS/Ch in place of HA are affected as well, due to the CS/Ch degradation activity of spider Hyals. Thus, spiders benefit most by having venom Hyal-like enzymes, which are able to degrade both, HA as well as CS. Therefore, despite being often called hyaluronidases, spider venom Hyal-like enzyme seems to be as important for CS degradation as they are for HA degradation.

## Supporting Information

S1 Fig MALDI-TOF-MS of purified CsHyal-like enzyme. Spectrum was recorded with purified CsHyal between 10–100 kDa in linear positive ion mode using sinapinic acid as matrix.
(DOCX)

S2 Fig The full set of annotated tandem mass spectra is given for all identified glycopeptide spectra of the glycosylation sites N134 and N360 of CsHyal. All spectra were manually interpreted and afterwards annotated by using R and the protViz CRAN package. (A) Annotated tandem mass spectra corresponding to the glycosylation site N134 represented by the glycopeptide AKELHPTANDSAVKEIAER. (B) Annotated tandem mass spectra corresponding to the glycosylation site N134 represented by the glycopeptide ELHPTANDSAVKEIAER. (C) Annotated tandem mass spectra corresponding to the glycosylation site N360 represented by the glycopeptide FYAGNITCR.
(DOCX)

S3 Fig Hyal activity and substrate specificity of different spider venoms. Degradation of GAG standards (HA, CS4, DS, and HS) after incubation with venom of the examined spider species.
(PDF)

S4 Fig Phylogenetic relationship between invertebrate and vertebrate Hyals. Vertebrates sequences are colored in different gray shades, arthropod sequences are colored in different brown shades. Mollusk and nematode sequences are not colored. Identical amino acid residues in all Hyals sequences are colored in red and identical amino acid residues to CsHyal sequence are colored in blue. The C-terminal EG-like domain is highlighted with yellow and position of amino acid residues responsible for the enzymatic activity of Hyals are marked with a black square. Putative disulfide bridges are connected by a black line; arachnid specific disulfides bridge in red line and, a comparable putative disulfide in the case of *C*. *elegans* by a dashed a red line.
(DOCX)

S1 Table Tabular summary of all identified glycopeptides on the glycosylation sites N134 and N360. (DOCX)
