# Supplementary material for: Isolation, N-glycosylations and Function of a Hyaluronidase-Like Enzyme from the Venom of the Spider Cupiennius salei
Source: PLoS One. 2015 Dec 2;10(12):e0143963. doi: 10.1371/journal.pone.0143963 (PMC4667920; doi:10.1371/journal.pone.0143963)

S1Figure. MALDI-TOF-MS of purified CsHyal-like enzyme. Spectrum was recorded with purified CsHyal between 10-100 kDa in linear positive ion mode

using sinapinic acid as matrix.


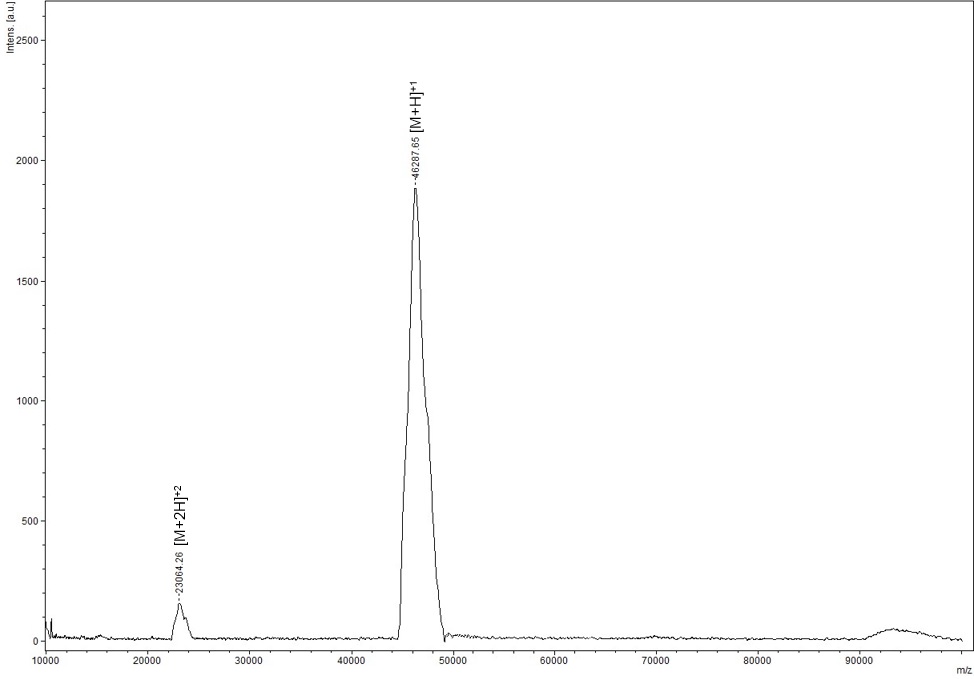

Supplement: S1 Fig — Spectrum was recorded with purified CsHyal between 10–100 kDa in linear positive ion mode using sinapinic acid as matrix. (DOCX) [file pone.0143963.s001.docx]
