# Supplementary material for: Isolation, N-glycosylations and Function of a Hyaluronidase-Like Enzyme from the Venom of the Spider Cupiennius salei
Source: PLoS One. 2015 Dec 2;10(12):e0143963. doi: 10.1371/journal.pone.0143963 (PMC4667920; doi:10.1371/journal.pone.0143963)
Supplement: S2 Fig — All spectra were manually interpreted and afterwards annotated by using R and the protViz CRAN package. (A) Annotated tandem mass spectra corresponding to the glycosylation site N134 represented by the glycopeptide AKELHPTANDSAVKEIAER. (B) Annotated tandem mass spectra corresponding to the glycosylation site N134 represented by the glycopeptide ELHPTANDSAVKEIAER. (C) Annotated tandem mass spectra corresponding to the glycosylation site N360 represented by the glycopeptide FYAGNITCR. (DOCX) [file pone.0143963.s002.docx]

S2 Figure. The full set of annotated tandem mass spectra is given for all identified glycopeptide spectra of the glycosilation sited N134 and N360 of CsHyal. All spectra were manually interpreted and afterwards annotated by using R and the protviz CRAN package.

1. Annotated tandem mass spectra corresponding to the glycosylation site N134 represented by the glycopeptide AKELHPTANDSAVKEIAER.

1. Annotated tandem mass spectra corresponding to the glycosylation site N134 represented by the glycopeptide ELHPTANDSAVKEIAER.

1. Annotated tandem mass spectra corresponding to the glycosylation site N134 represented by the glycopeptide FYAGNITCR.
