# Supplementary material for: Isolation, N-glycosylations and Function of a Hyaluronidase-Like Enzyme from the Venom of the Spider Cupiennius salei
Source: PLoS One. 2015 Dec 2;10(12):e0143963. doi: 10.1371/journal.pone.0143963 (PMC4667920; doi:10.1371/journal.pone.0143963)
Supplement: S3 Fig — Degradation of GAG standards (HA, CS4, DS, and HS) after incubation with venom of the examined spider species. (PDF) [file pone.0143963.s003.pdf]

S3 Figure. Hyal activity and substrate specificity of different spider venoms. Degradation of GAG standards (HA, CS4, DS, and HS) after incubation with venom of the examined spider species. Standards were incubated in a ratio of 1:1 with 1:25 diluted venom in 200 mM NaAc, 150 mM NaCl, pH 5.8 for HA, CS4, and DS, and in a 50 mM Tris-HCl, 150 mM NaCl buffer, pH 7.5 for HS. The degradation process was revealed after agarose gel electrophoresis and sequential staining with toluidine blue and Stains-All.

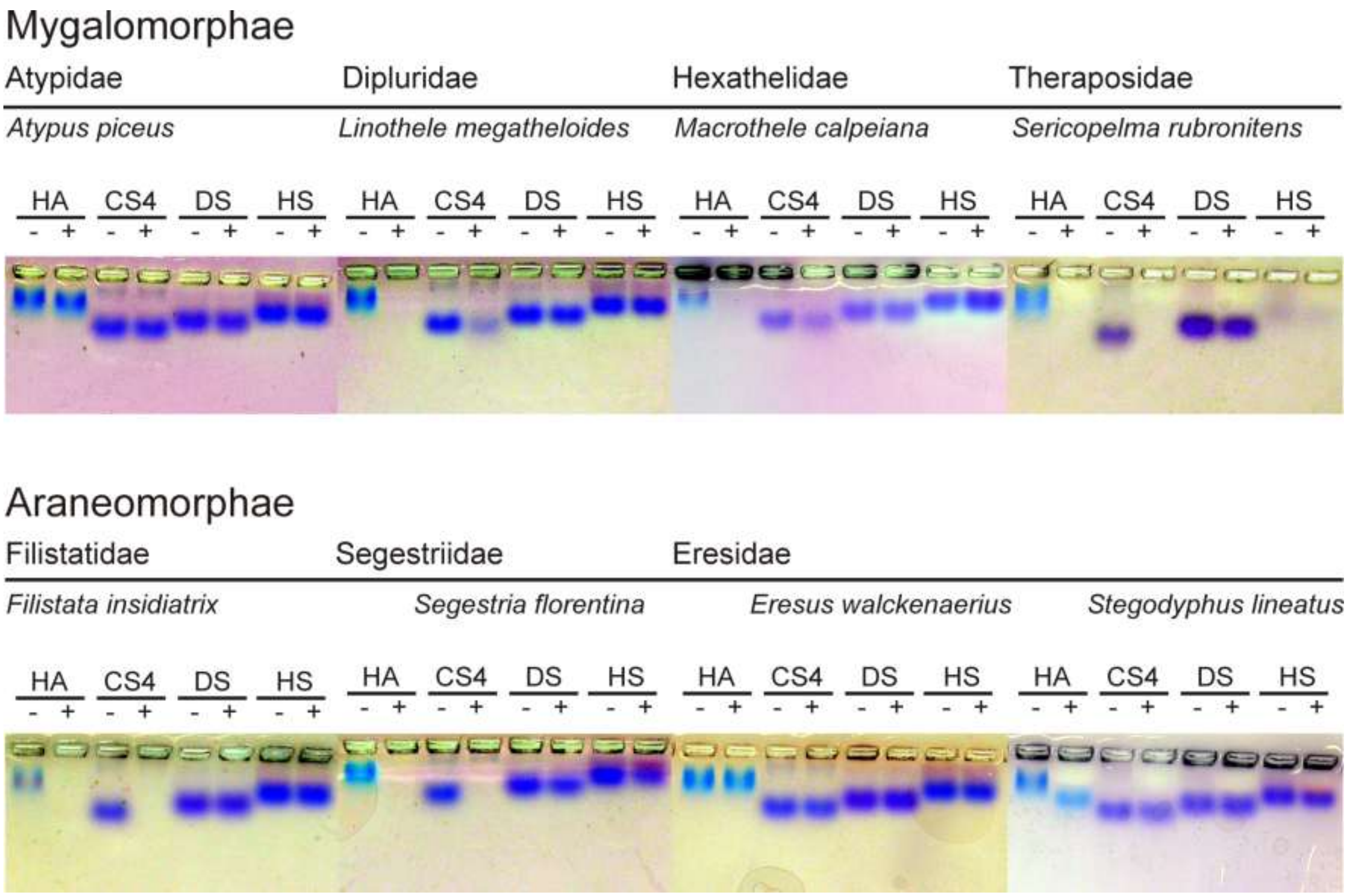

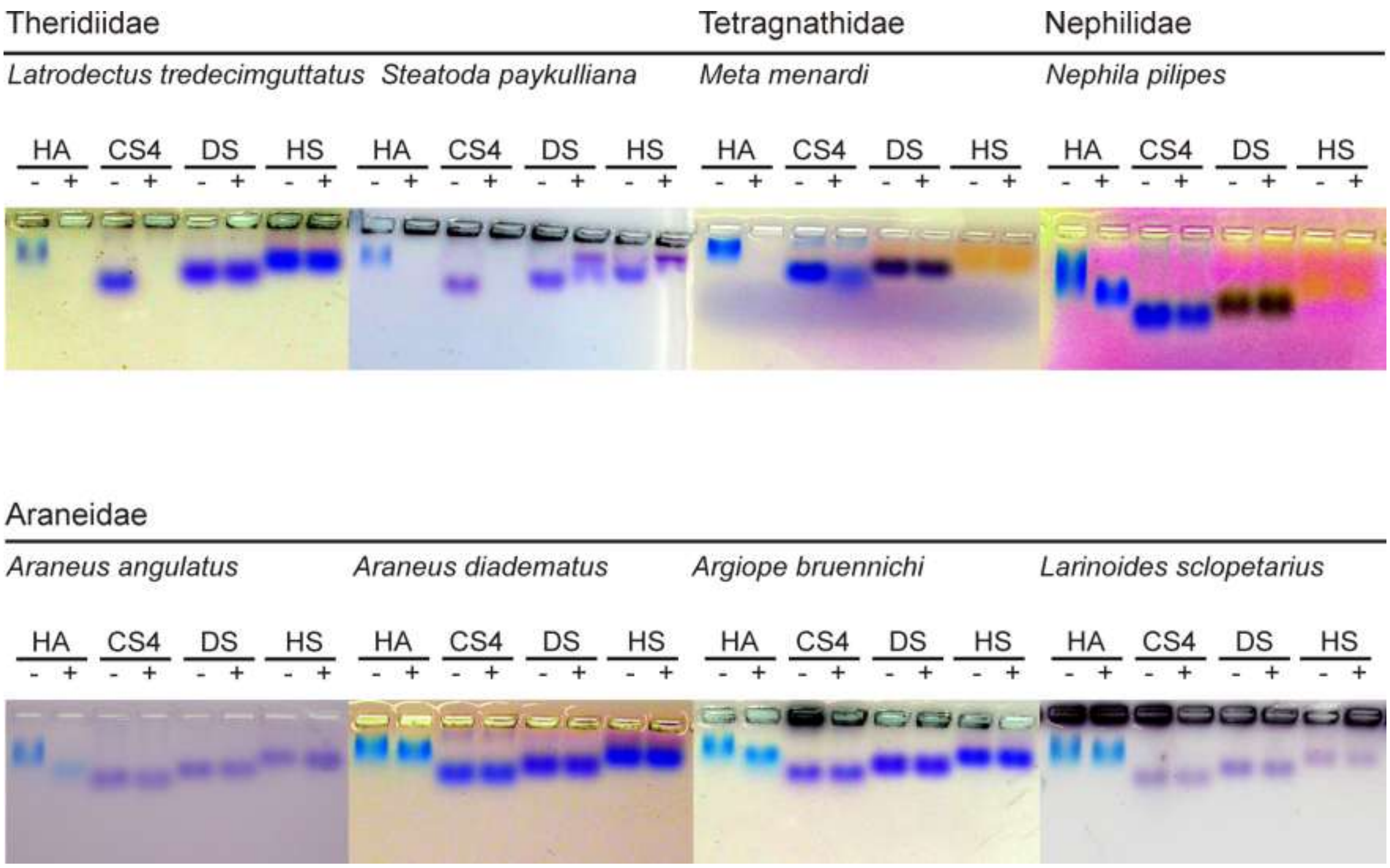

Theridiidae
Tetragnathidae
Nephilidae
Latrodectus tredecimguttatus
Steatoda paykulliana
Meta menardi
Nephila pilipes
HA CS4 DS HS HA CS4 DS HS HA CS4 DS HS HA CS4 DS HS
- + - + - + - + - + - + - + - + - + - + - + - + - + - + - + - +
Araneidae
Araneus angulatus
Araneus diadematus
Argiope bruennichi
Larinoides sclopetarius
HA CS4 DS HS HA CS4 DS HS HA CS4 DS HS HA CS4 DS HS
- + - + - + - + - + - + - + - + - + - + - + - + - + - + - + - +

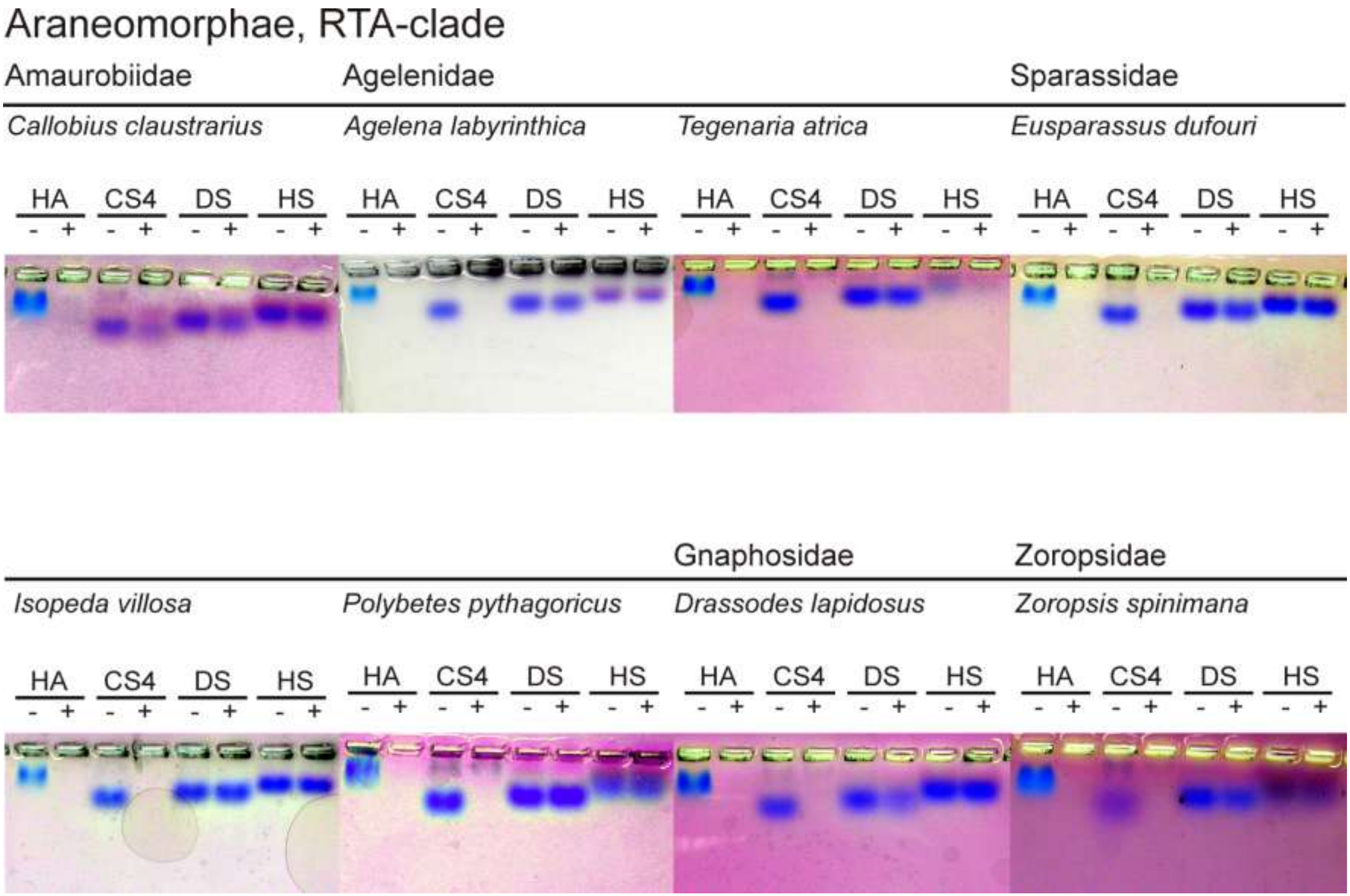

Araneomorphae, RTA-clade
Amaurobiidae
Agelenidae
Sparassidae
Callobius claustrarius
Agelena labyrinthica
Tegenaria atrica
Eusparassus dufouri
HA CS4 DS HS
- + - + - + - +
Gnaphosidae
Zoropsidae
Isopeda villosa
Polybetes pythagoricus
Drassodes lapidosus
Zoropsis spinimana
HA CS4 DS HS
- + - + - + - +

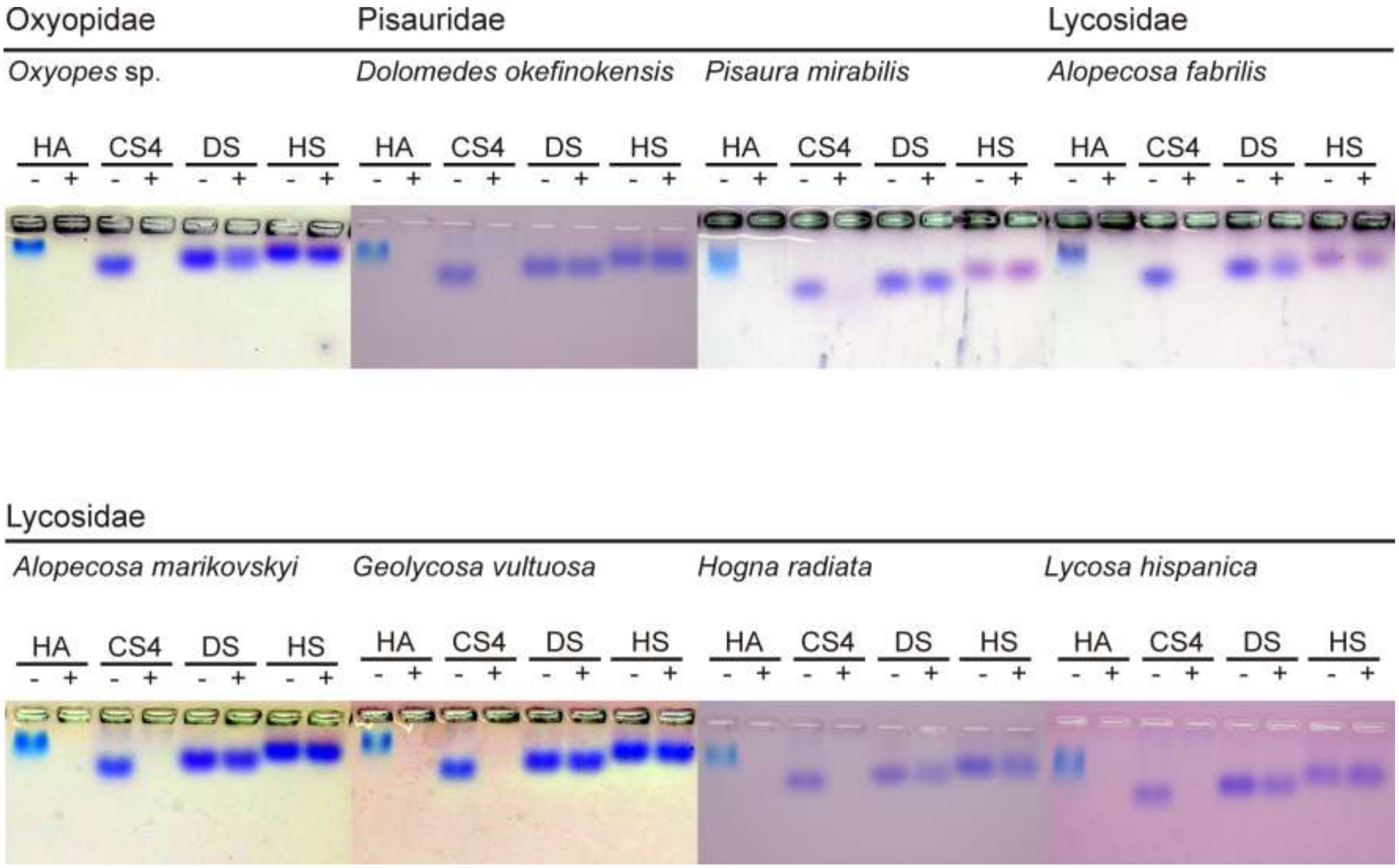
Oxyopidae
Pisauridae
Lycosidae
Oxyopes sp.
Dolomedes okefinokensis
Pisaura mirabilis
Alopecosa fabrilis
HA CS4 DS HS HA CS4 DS HS HA CS4 DS HS HA CS4 DS HS
- + - + - + - + - + - + - + - + - + - + - + - + - + - + - + - +
Lycosidae
Alopecosa marikovskyi
Geolycosa vultuosa
Hogna radiata
Lycosa hispanica
HA CS4 DS HS HA CS4 DS HS HA CS4 DS HS HA CS4 DS HS
- + - + - + - + - + - + - + - + - + - + - + - + - + - + - + - +

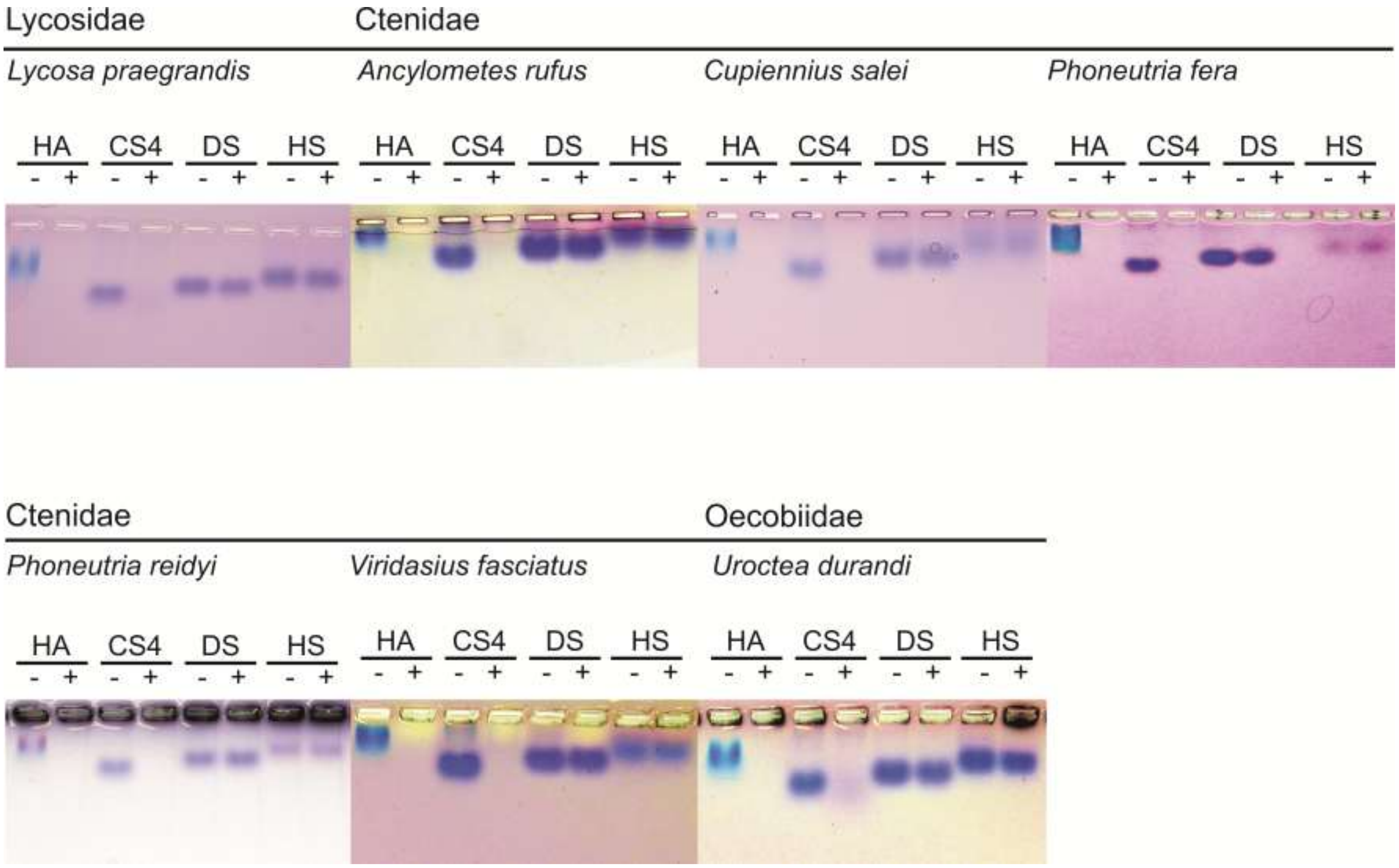
Lycosidae
Ctenidae
Lycosa praegrandis
Ancylometes rufus
Cupiennius salei
Phoneutria fera
HA CS4 DS HS
- + - + - + - +
Ctenidae
Oecobiidae
Phoneutria reidyi
Viridasius fasciatus
Uroctea durandi
HA CS4 DS HS
- + - + - + - +
