# Supplementary material for: Isolation, N-glycosylations and Function of a Hyaluronidase-Like Enzyme from the Venom of the Spider Cupiennius salei
Source: PLoS One. 2015 Dec 2;10(12):e0143963. doi: 10.1371/journal.pone.0143963 (PMC4667920; doi:10.1371/journal.pone.0143963)
Supplement: S4 Fig — Vertebrates sequences are colored in different gray shades, arthropod sequences are colored in different brown shades. Mollusk and nematode sequences are not colored. Identical amino acid residues in all Hyals sequences are colored in red and identical amino acid residues to CsHyal sequence are colored in blue. The C-terminal EG-like domain is highlighted with yellow and position of amino acid residues responsible for the enzymatic activity of Hyals are marked with a black square. Putative disulfide bridges are connected by a black line; arachnid specific disulfides bridge in red line and, a comparable putative disulfide in the case of C. elegans by a dashed a red line. (DOCX) [file pone.0143963.s004.docx]

S4 Figure. Phylogenetic relationship between invertebrate and vertebrate Hyals (Clustal Omega (http://www.ebi.ac.uk/Tools/services)

**↓ Start mature sequence** ■

*C. consors* -------------------------------------------------MRAV--V---VVTGLVVVVVATALSLPNHDVKSATSSRSSSDYQGSSGDDCDEGLPPPDQPFRVV**W**NHP-DN**C**ERIKLHLP---LDDYGIIFNKLRVFLG-EEIQTL**Y**DT--**G**PW**P**YISE--TGK---FIN**G**GLP**Q**SFNHPDNDGETQRILKKHRP-ENFT

*S. horrida* ----------------------------------------------------MIKLKFLYVQHF-FSISVTMMSLLR----------------SGGALPWTDPPLHPGHPFLFT**W**NAPTEL**C**GIRFGMPLD--LSYFDFVSSTLKSATN-QSISIF**Y**TDRF**G**VF**P**YVNEK-TGK---MYN**G**GLP**Q**LIDLEQHHELAEDDIEYYIPFN-QL

*C. adamanteus* ------------------------------------------------------------MYHL-WIKCL-AAWIFLKRFNGV-------------HVMQAKAPMYPNEPFLVF**W**NAPTTQ**C**RLRYKVDLD--LNTFHIVTNANDSLSG-SAVTIF**Y**PTHL**G**FY**P**HIDG--RGH---FFN**G**IIP**Q**NESLAKHLNKSKSDINRMIPLRTFH

*HUMAN Hyal-1* -----------------------------------------------------------MAAHL-LPICALFLTLLD-------------------MAQGFRGPLLPNRPFTTV**W**NANTQW**C**LERHGVDVD--VSVFDVVANPGQTFRG-PDMTIF**Y**SSQL**G**TY**P**YYTP--TGE---PVF**G**GLP**Q**NASLIAHLARTFQDILAAIPAPDFS

*X. laevis* -----------------------------------------------------------------MPCCLTFLWLFLGAAANA-------QLSDSWMNKPTFRPVFTRRPFIIA**W**NAPTQD**C**PPRFDVHLD--LKLFDLNASPNEGFVD-QNLTIF**Y**KERL**G**MY**P**YYDE--HGG---PVA**G**GLP**Q**NASLRAHLDKLPEGIQKYIRSRDRD

*T. castaneum* ---------------------------------------------------------MSLIFALFILLRFLFTIMESGTFESN---------------EILAKKLTKVQKVNYY**W**NVPTFQ**C**D-SHKLNFTGLADKFGIIQNENDRFRG-NEVAIL**Y**D--P**G**SF**P**ALLRDGSGIV--RRN**G**GVP**Q**EGNLTLHLALFEELLNELLP-EDFS

*A. echinatior* MSIDAHQAQYSYSRAHTCPLYCGIIISKKREKTRRGIRAKKRKRRIIEQYPEPDNTTRRGRTGVS----KKDDKL-LGNSWG-----------------TTTLQASNSRQFDVY**W**NVPSFM**C**N-QYNVRFDDLK-DFGIHQNTMDEFRG-EEIAIL**Y**D--P**G**MF**P**ALLTDKTGTVTNVRN**G**GVP**Q**EGDLKKHLEMFQKHLIKQIPDGSFS

*A. mellifera* -------------------------------------------------MSRPLVITEGMMIGVLLMLAPINALL-LG-FVQ-----------------STPDNNKTVREFNVY**W**NVPTFM**C**H-KYGLRFEEVSEKYGILQNWMDKFRG-EEIAIL**Y**D--P**G**MF**P**ALLKDPNGNVV-ARN**G**GVP**Q**LGNLTKHLQVFRDHLINQIPDKSFP

*V. vulgaris* -------------------------------------------------------------------------------------------------------SERPKRVFNIY**W**NVPTFM**C**H-QYDLYFDEVT-NFNIKRNSKDDFQG-DKIAIF**Y**D--P**G**EF**P**ALLSLKDGKYK-KRN**G**GVP**Q**EGNITIHLQKFIENLDKIYPNRNFS

*C. elegans* ----------------------------------------------------M--VIVWYHQLL-----LVL-LIFIGAAKGA---------------QYIGSGASQPNRTDVV**W**MVPSWT**C**KNEYSID----VEKYGILQNEDQHFVGGKQFAIF**Y**EHSF**G**KI**P**YFKAQNESD---PKN**G**GLP**Q**MGDLEAHLIQAEKDINETIPDENFN

*S. maritima* ------------------------------------------------MFPRE--IFLWTFYLF-----LSRFETSL-------------------------ALIEPQHDFIVI**W**NVPTQK**C**SK---LNFTFDLSKYNIIHNENGSFDG-EKITIF**Y**K--I**G**KF**P**SISNSG--E---YIN**G**GLP**Q**LGQFSDHLDQVKEDVTDAIPDAEFN

*M. martensii* -------------------------------------------------MTQN--IQMTEMYQI-----ILFASILA-------------------------AISATSADFKVV**W**EVPSIM**C**SKKFKINVTDLLTSHKILVNQEETFNG-DKIVIF**Y**ESQL**G**KY**P**HIESH---G---DIN**G**GML**Q**VSDLANHLKIARDNISKFIPDPNFN

*T. serrulatus* ------------------------------------------------------------------------------------------------------------ADFKVY**W**EVPSFL**C**SKRFKINVTEVLTSHEILVNQGESFNG-DKIVIF**Y**ENQL**G**KY**P**HIDSNN--V---EIN**G**GIL**Q**VADLAKHLKVAKDNITKFVPNPNFN

*C. salei* ------------------------------------------------------------MET--------R-IIFL-------------------------HLLAIVSGFKIY**W**NVPTFQ**C**THNYKIDYVKLLSTYGIQVNDGGKFQG-NQVTIF**Y**ETQL**G**LY**P**RILKSG--K---MEN**G**GIP**Q**RGNFEKHLEKASTDLQKVIPWKEFS

*L. intermedia* ------------------------------------------------------------MQTI-----LVL-TTFL-------------------------SAWFLAVGFDVF**W**NVPSQQ**C**K-KYGMKFVPLLEQYSILVNKEDNFKG-DKITIF**Y**ESQL**G**LY**P**HIGAND--E---SFN**G**GIP**Q**LGDLKAHLEKSAVDIRRDILDKSAT

*B. vagans* ---------------------------------------------------------------------------------------------------------KDPQVFAVR**W**NVPTIQ**C**RKTYGMDFVPLLKSYGILVNSEDEFKG-EVNTIF**Y**EGQL**G**LY**P**HLDQSG--Q---RVN**G**GIP**Q**LGDLPEHLKKAREDINKAIPDVNFN

■ ■ ■ ■ ■

*C. consors* **G**LGVL**D**F**E**TWRAIYSTNFGPMTIYQNESVKLVKEQHPDYD-QKKLTKVAEKEWQQAAKDIMSNKLKIAQEVMPRGH**W**GY**Y**LY**P**RTWDNKRD-------------TKFR**N**DKIN**WL**WRQSTGLYPSI**Y**IYDF-S-K---TESAITKFVSDTVG**E**AV**R**VQKEFSP--PNTPIYPYVMFQTM--DNIFHYEDHLKISLGLSAKMGAAGVVL**W**G

*S. horrida* **G**LAVL**D**F**E**EWRPQWIRNWGSKDIYRQYSIETVLKKNSSLS-KEEAAGQAKMAFERAAKKYFLRSIRIGKRLRPNRL**W**GY**Y**LY**P**E**C**YNYEYKKDMAGYTGE**C**PAIEKDR**N**NELL**WL**WRESTALFPSI**Y**LELL-L-R---DTQQARQYVRHRIQ**E**SI**R**VSKL-PNSAYSIPIHAYVRPVYKDSTDNYMSEFDLVNTIGEAAALGAASVVC**W**G

*C. adamanteus* **G**LGVI**D**W**E**NWRPQWDRNWGSKNVYRNRSIQFARDLHPELS-EDEIKRLAKQEYEKAAKSFMRDTLLLAEEMRPYGY**W**GY**Y**LY**P**D**C**QNYNYKTKPDQYTGE**C**PDIEITR**N**NQLL**WL**WRDSTALFPNI**Y**LETV-L-R---SSDNALKFVHHRLK**E**SM**R**IASM-ARKDYALPVFPYARPFYAY-TFEPLTEEDLVNTVGETAAMGAAGIVF**W**G

HUMAN Hyal-1 **G**LAVI**D**W**E**AWRPRWAFNWDTKDIYRQRSRALVQAQHPDWP-APQVEAVAQDQFQGAARAWMAGTLQLGRALRPRGL**W**GF**Y**GF**P**D**C**YNYDFLS--PNYTGQ**C**PSGIRAQ**N**DQLG**WL**WGQSRALYPSI**Y**MPAV-L-E---GTGKSQMYVQHRVA**E**AF**R**VAVA--AGDPNLPVLPYVQIFYDT-TNHFLPLDELEHSLGESAAQGAAGVVL**W**V

*X. laevis*  **G**LAVI**D**W**E**EWRPIWMRNWQTKNVYRNNSRNLVASRHPSWS-REQVEKESLYDFENAAREFMMETLRHAKTTRPRQL**W**GF**Y**LF**P**D**C**YNHDYIKNRESYTGQ**C**PDVEISR**N**DQLS**WL**WEKSTALYPSI**Y**LGQV-L-R---RLRTGRKFVRSRVR**E**AM**R**ISYR-HHKDYSLPVFVYTRPTYIR-KLDFLSQMDLISTIGESAAQGAAGVIF**W**G

*T. castaneum* **G**LGII**D**F**E**SWRPIYRQNFGSLAPYKDLSVEIERQSHPFWP-KLLLEKEARRRFEFHARRFMEETLFVAKSLRKNAT**W**GY**Y**AY**P**Y**C**FNMSPN----NMKKD**C**PNEVQKE**N**DQLD**WL**FRLSDDLHPSI**Y**LDGR-L-----GPKDKIRMIEGRIN**E**AH**R**VAAFVKSGALKPKIVPYFWYKYHGGQ-NFLTKEDLFNAILTLSTSDIDGVVI**W**G

*A. echinatior* **G**IGVI**D**F**E**SWRPIFRQNWASLEPYKTLSLKLEHKRHPFWS-ESATKKEAKRRFEKYARIFMEETLNMAKKLRSKAK**W**GY**Y**GY**P**Y**C**FNQTPG----QPTAH**C**NRQTMAE**N**NEMS**WL**FTLEDVHLSSV**Y**LRQE-I-----RGEDRVGFVKGRVS**E**AL**R**MAGKIPR--K-QQVLPYYWFKYQDNRDNFLSEKDTENTFNTIANLGADGLII**W**G

*A. mellifera* **G**VGVI**D**F**E**SWRPIFRQNWASLQPYKKLSVEVVRREHPFWD-DQRVEQEAKRRFEKYGQLFMEETLKAAKRMRPAAN**W**GY**Y**AY**P**Y**C**YNLTPN----QPSAQ**C**EATTMQE**N**DKMS**WL**FESEDVLLPSV**Y**LRWN-L-----TSGERVGLVGGRVK**E**AL**R**IARQMTT--SRKKVLPYYWYKYQDRRDTDLSRADLEATLRKITDLGADGFII**W**G

*V. vulgaris* **G**IGVI**D**F**E**RWRPIFRQNWGNMKIHKNFSIDLVRNEHPTWN-KKMIELEASKRFEKYARFFMEETLKLAKKTRKQAD**W**GY**Y**GY**P**Y**C**FNMSPN----NLVPE**C**DVTAMHE**N**DKMS**WL**FNNQNVLLPSV**Y**VRQE-L-----TPDQRIGLVQGRVK**E**AV**R**ISNNLKH--S-PKVLSYWWYVYQDETNTFLTETDVKKTFQEIVINGGDGIII**W**G

*C. elegans* **G**IAVI**D**I**E**EFRPMWELSWGPFSVYKTESIRLTRQQHPYWS-TKQIEWQAERDYEKA**C**QKFFIETLRLGKRLRPNAK**W**GY**Y**LF**P**K**C**NGDVGQ----KSDTD**C**STLFQKF**N**DNLH**WL**WGESTALFPSI**Y**LYPSQKQNPEYNFVNSG----ALIT**E**TK**R**IKRNY**C**PS-**C**EIHVFTKIEYNPYYTPDDFYSKQNLASTLDLAIKMNANSVVI**W**S

*S. maritima* **G**FAVI**D**W**E**SWRPTFQYNWGELNKYKEESRNLVKKGHLLSNTTFEIETRAEFEFEIAARLYMQYTINISQTLRPRGN**W**GY**Y**GF**P**D**C**YNYEHL------EKY**C**SNDIQHY**N**DKTS**WL**FNSSSVLYPSI**Y**ISKNNITEN-------SVRIYGKLY**E**AN**R**VG----Q---KLAIYPYAIVHYQD-GTDFLSAEDLLITIGQAGGLRFPGIVL**W**G

*M. martensii* **G**VGII**D**W**E**AWRPLWKYNWGRMSEYRDRSKDLVKAKHPDWS-PAQIEKVAIEEWENSAKEWMLKTLKLVEDMRPNAA**WC**Y**Y**LF**P**D**C**YNYGGKD--QPSEYF**C**KNDIQEA**N**DKLS**WL**WKQSTAL**C**PSI**Y**MQESHITKY--NTSQRAWWIYARLR**E**TI**R**LS---HP---NTLIYPYINYILPG-TKKTVPSMDFKRVLGQIGSLGLDGAII**W**G

*T. serrulatus* **G**VGVI**D**W**E**AWRPSWEFNWGKLKVYKEKSIDLVKSKHPEWP-SDRVEKVAKEEWEESAKEWMVKTLKLAQEMRPNAV**WC**Y**Y**LF**P**D**C**YNYFGKD--QPSQFS**C**SSRIQKE**N**SRLS**WL**WNQSTAI**C**LSI**Y**IQESHVTKY--NMSQRTWWIDARLR**E**AV**R**VS-EHRP---NIPIYPYINYILPG-TNQTVPAMDFKRTLGQIASLGLDGALL**W**G

*C. salei* **G**LGVI**D**W**E**AWRPTWEFNWEPLRIYQTESIKRAKELHPTAN-DSAVKEIAERQWEDSAKLYMLETLRLAKKLRPQAP**WC**Y**Y**LF**P**D**C**YNYVGK---KPKDFQ**C**SASIRKG**N**DKLS**WL**WKDSTAL**C**PSI**Y**VYESQLDRY--SFEQRTWRDNEKLR**E**AL**R**VA---TR---TSKIYPYVNYF----DKELIPEQEVWRMLAQAAAVGGSGAVI**W**G

*L. intermedia* **G**LRII**D**W**E**AWRPIWEFNWSSLRKYQDKMKKVVRQFNPTAH-ESTVAKLAHNEWENSSKSWMLSTLQLGKQLRPNSV**WC**Y**Y**LF**P**D**C**YNYDGN---SVQEFQ**C**SEAIRKG**N**DRLK**WL**WEESTAV**C**PSI**Y**IKEGQLTNY--TLQKRIWFTNGRLQ**E**AL**R**VA---QP---KARIYPYINY--SIKPGMMVPEVEFWRLIAQIASLGMDGAVI**W**G

*B. vagans* **G**LGII**D**W**E**SWRPVWNFNWGALKKYQDESFEEALKQHPGRT-NDSLWQLAQQEWETSAKNFMLETLRLAQTMRPNSL**WC**Y**Y**LF**P**D**C**YNYNGQ---TPQEFR**C**PSIVVTG**N**NQLS**WL**WHESKAV**C**PSL**Y**VADGYLQKY--TFEQRTWYVDGRLK**E**AL**R**VA----P---NSQLYPYVGYGYGVTPGAMVPEDDFWRILAQVASAGSSGTVI**W**G

**? ?**

*C. consors* TSKHYKESTRQWQ**C**QQLQEHIRTVLGPLVKNVTQMMTD**C**SRAI**C**EGHGR**C**VHNSHDVIL-GET--ES--------------QRLSDLCST--RQSRFRDYH**C**R**C**YSAWEGA**CC**QTLRPSRCQKREQRNVHGGGDLID

*S. horrida* DMSVVAT---EDS**C**FDARRHLEKVMNPYIMNVSTATQL**C**SKAL**C**QDQGR**C**VRKHWDDDVFLHLDPRRYRIEQQRG---GGPLTVTGDLSQDDVNWFDRNFD**C**M**C**YSEKP**C**RSALTFNVINKAVINKAPPACDGTARSDVHCDVNINF

*C. adamanteus* SMQYAST---VDS**C**RKVKDYIDGPLGRYIVNVTTAAKI**C**SHFL**C**KKHGR**C**VRKHSDSNAFLHLFPDSFRILVHG-NATEKKVIVKGKLELENLIFLINNFM**C**Q**C**YQGWKGLY**C**EKHSIKDIRKI

*HUMAN Hyal-1* SWENTRT---KES**C**QAIKEYMDTTLGPFILNVTSGALL**C**SQAL**C**SGHGR**C**VRRTSHPKALLLLNPASFSIQLTPG---GGPLSLRGALSLEDQAQMAVEFK**C**R**C**YPGWQAPW**C**ERKSMW-

*X. laevis* DAEYTKS---KET**C**QMIKRYLEEDLGRYIVNVTTAAEL**C**SQSL**C**NGNGR**C**LRQDNITDAFLHLNSANFQIVSAPKDSQGPSLRAEGKLSAEDIAVLRSQFR**C**Q**C**YVDWYGDS**C**GFQRRTNGGAVATGPCGIVLVVSLVALILALL

*T. castaneum* SSNDVNT---KNK**C**LDLYEYVDNVLGPSLVNF----------------------------------------------------------------------------------------

*A. echinatior* SSEDTNT---EQK**C**KDLLHYVRTILGPAIKRIK---------------------------------------------------------------------------------------

*A. mellifera* SSDDINT---KAK**C**LQFREYLNNELGPAVKRIALNNNANDRLTVDVSVDQV---------------------------------------------------------------------

*V. vulgaris* SSSDVNS---LSK**C**KRLQDYLLTVLGPIAINVTEAVN-----------------------------------------------------------------------------------

*C. elegans* TSQSIGS-----R**C**GSLQTYVDNTLGPYLQLTDRNLDK**C**RMER**C**EGRGE**C**YLPRPKTNP------------------------------------AIYNFA**C**R**C**ERPYFGKS**C**EYRGRRMGVSMPKASQTPQVIPDVTAYFSTSSNGTKKYNAPNQFYSRTGGDIKLARKL

*S. maritima* SSSTLGS---KEK**C**EEFSIYFNTILGPVLNKIRSVLKN-------KTESLQFPDSLDPE-------LWAE--------------------KIIQFYQ--------------------------------------------------------------------------

*M. martensii* SSYHVNT---EEM**C**KEMKTYVKDVIAPVASTVIQNVNR**C**SQQI**C**KGRGN**C**VWPEEPY-T-------SWKY----------------LIDPKNPTFKHTNIS**C**K**C**KGGYTGRY**C**QIAP------------------------------------------------------

*T. serrulatus* SSYHVLT---ESQ**C**KITSDYVKSVIAPTVATVVLNTNR**C**SQII**C**KGRGN**C**VWPEEPF-S-------SWKY----------------LVDPKMPVFKPTNIH**C**K**C**K-GYLGRY**C**EIPK------------------------------------------------------

*C. salei* SSAAVAS---EEL**C**KSLKQYIIETLGPAAEKVAWRSDL**C**SKEI**C**NNQGR**C**TFPDDDYAN-------AWKL----------------FTD-DTVKFYAGNIT**C**R**C**SENYSGRF**C**EKKN------------------------------------------------------

*L. intermedia* SSASVGS---KNH**C**AQLMKYIADVLGPATLRIKENVAR**C**SKQA**C**SGRGR**C**TWPKDTSVI-------AWKF----------------LVEKEDYDFYLGDIE**C**K**C**VEGYEGRY**C**EQKTK-----------------------------------------------------

*B. vagans* ASATLRS---RDN**C**QLLQQYVKDILGPSVKTVKENAER**C**AKTV**C**NGKGR**C**TWPNDPNVV-------AWRV----------------YLDRNKHPFQRSEIT**C**H**C**VEGYSGRY**C**DVKSGITNQTKRVSFK----LSELYTYLRRLLDN------------------------

EGF-like domain prediction InterPro EGF-like site prediction InterPro

**X** Identical amino acid residues in all sequences excluding signal& propeptide

X identical amino acid residues to CsHyal excluding signal& propeptide

X assumed EGF-like site/domain (UniProt.org/InterPro)

**C-C disulphid bridges**

**C-C disulphid bridges only in arachnids and nematodes**

■ important amino acid residues responsible for the enzymatic activity of Hyals ■ proton donor

**Species % identity to *Cupiennius salei* Accession numbers** S4 Figure. Phylogenetic relationship between invertebrate and vertebrate Hyals. Vertebrates

*Conus consors*  invertebrate mollusk 31.1 I0CME7 sequences are colored in different gray shades, arthropod sequences are colored in different brown shades.

*Synanceia horrida*  vertebrate fish 30.2 Q801Z8 Mollusk and nematode sequences are not colored. Identical amino acid residues in all Hyals sequences are

*Crotalus adamanteus*  vertebrate snake 36.4 J3S820 colored in red and identical amino acid residues to CsHyal sequence are colored in blue. The C-terminal

*Homo sapiens*  vertebrate mammal 33.9 Q12794 EG-like domain is highlighted with yellow and position of amino acid residues responsible for the

*Xenopus laevis*  vertebrate amphibian 30.5 Q8UVY6 enzymatic activity of Hyals are marked with a black square. Putative disulfide bridges are connected by a

*Tribolium castaneum*  invertebrate insect 34.4 D2A580 black line; arachnid specific disulfides bridge in red line and, a comparable putative disulfide in the case

*Acromyrmex echinatior*  invertebrate insect 35.0 F4WSI6 of *C. elegans* by a dashed a red line.

*Apis mellifera*  invertebrate insect 34.4 Q08169

*Vespa vulgaris*  invertebrate insect 36.7 P49370

*Caenorhabditis elegans*  invertebrate nematode 35.9 G5ECE8

*Strigamia maritima*  invertebrate centipede 36.9 T1JN23

*Mesobuthus martensii*  invertebrate scorpion 42.1 P86100

*Tityus serrulatus*  invertebrate scorpion 41.8 P85841

*Cupiennius salei*  invertebrate spider 100.0

*Loxosceles intermedia*  invertebrate spider 49.9 R4J7Z9

*Brachypelma vagans*  invertebrate spider 49.5 J9XYC6
