# Supplementary material for: Isolation, N-glycosylations and Function of a Hyaluronidase-Like Enzyme from the Venom of the Spider Cupiennius salei
Source: PLoS One. 2015 Dec 2;10(12):e0143963. doi: 10.1371/journal.pone.0143963 (PMC4667920; doi:10.1371/journal.pone.0143963)
Supplement: S1 Table — (DOCX) [file pone.0143963.s005.docx]

S1 Table. Tabular summary of all identified glycopeptides on the glycosylation sites N134 and N360.

1. Glycosite N360

1. Glycosite N134 peptide ELHPTANDSAVKEIAER (fully tryptic)

1. Glycosite N134 peptide AKELHPTANDSAVKEIAER (one missed cleavage)
